# AIM2 controls microglial inflammation to prevent experimental autoimmune encephalomyelitis

**DOI:** 10.1084/jem.20201796

**Published:** 2021-03-12

**Authors:** Chunmei Ma, Sheng Li, Yingchao Hu, Yan Ma, Yuqing Wu, Chunyan Wu, Xue Liu, Bingwei Wang, Gang Hu, Jiawei Zhou, Shuo Yang

**Affiliations:** 1 Department of Immunology, Key Laboratory of Immunological Environment and Disease, State Key Laboratory of Reproductive Medicine, Center for Global Health, Nanjing Medical University, Nanjing, China; 2 Department of Pharmacology, Nanjing University of Chinese Medicine, Nanjing, China; 3 Institute of Neuroscience, State Key Laboratory of Neuroscience, Center for Excellence in Brain Science and Intelligence Technology, Shanghai Institutes for Biological Sciences, Chinese Academy of Sciences, Shanghai, China; 4 School of Future Technology, University of Chinese Academy of Sciences, Beijing, China

## Abstract

The role of the PYHIN family member absent in melanoma 2 (AIM2), another important inflammasome sensor, in EAE remains unclear. In this study, we found that AIM2 negatively regulates the pathogenesis of EAE independent of inflammasome activation. AIM2 deficiency enhanced microglia activation and infiltration of peripheral immune cells into the CNS, thereby promoting neuroinflammation and demyelination during EAE. Mechanistically, AIM2 negatively regulates the DNA-PK–AKT3 in microglia to control neuroinflammation synergistically induced by cGAS and DNA-PK. Administration of a DNA-PK inhibitor reduced the severity of the EAE. Collectively, these findings identify a new role for AIM2 in controlling the onset of EAE. Furthermore, delineation of the underlying inflammasome-independent mechanism highlights cGAS and DNA-PK signaling as potential targets for the treatment of heterogeneous MS.

## Teaser

In this study, Ma et al. demonstrate that AIM2 negatively regulates the DNA-PK–AKT3 in microglia to control neuroinflammation synergistically induced by cGAS and DNA-PK, thereby preventing the pathogenesis of EAE independent of inflammasome activation.

## Graphical Abstract

**Figure:**
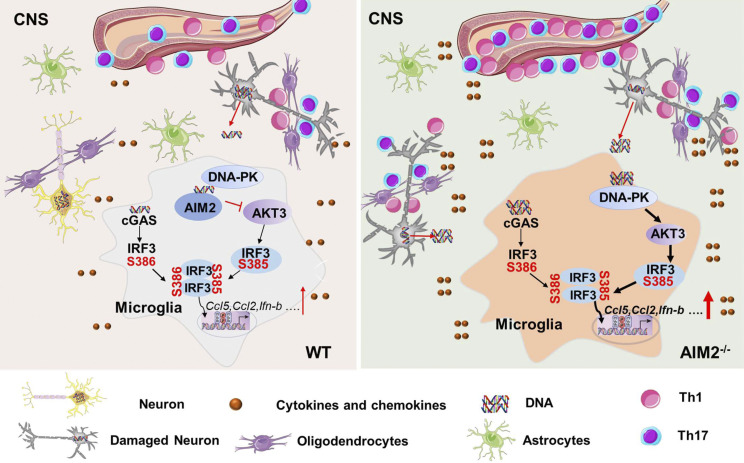

## Introduction

Multiple sclerosis (MS) is a chronic inflammatory autoimmune disease of the central nervous system (CNS) and affects ∼2.5 million people globally. MS causes the dysfunction in motor, sensory, visual, and autonomic systems of patients, and its neuropathological features include inflammatory cell infiltration, chronic axonal damage, and demyelination of the CNS (Dendrou et al., 2015). As a multifactorial, heterogeneous disease, MS is difficult to treat. Thus, the etiology and underlying basis of this heterogeneous disease remain largely unknown.

Experimental autoimmune encephalomyelitis (EAE) is a commonly used experimental animal model of MS study and recapitulates many of neuropathological features of MS (Ransohoff, 2012). Myelin oligodendrocyte glycoprotein (MOG)–induced EAE in C57BL/6 mice is often used to explore multiple facets of the mechanism surrounding immune-mediated demyelination, especially in transgenic/KO mice (Constantinescu et al., 2011; Procaccini et al., 2015). Front-line clinical treatments for MS including IFN-β, glatiramer acetate, anti–VLA-4 integrin mAb, and fumaric acid esters have been developed, tested, and validated in the MOG-induced EAE model (Aharoni et al., 2008; Galligan et al., 2010; Humphries et al., 2020; Mindur et al., 2014). Such models can be induced in different ways through innate immune activation. Low doses of the adjuvant (mycobacteria [Mtb]) are able to induce a subtype of EAE termed type A, which is NLRP3 inflammasome dependent. In contrast, high doses of Mtb or acute virus infection can induce a subtype of EAE termed type B, which occurs independently of NLRP3 inflammasome (Inoue et al., 2016). Thus, to elucidate the key molecules and underlying mechanisms involved in the distinct MOG-EAE subtypes may provide new insights into developing diagnostic indicators and treatments for heterogeneous MS.

NLRP3 is assembled into multimeric complexes with apoptosis-associated speck-like protein containing CARD (ASC) and pro-inflammatory caspases (caspase-1 and -11) known as an inflammasome. Inflammasome assembly leads to caspase autoactivation and the subsequent cleavage of pro–IL-1β and pro–IL-18 precursors into their mature forms. Inflammasome activation also results in an inflammatory form of cell death known as pyroptosis (Rathinam et al., 2012). Several independent studies have reported a critical role for the NLRP3 inflammasome and its related proteins such as ASC and gasdermin D in the development of type A EAE (Inoue et al., 2012; Li et al., 2019b; Martin et al., 2016). Absent in melanoma 2 (AIM2) is a PYHIN (pyrin and HIN domain containing) family member and an important inflammasome sensor that detects cytosolic double-stranded DNA via its HIN200 domain (Hornung et al., 2009). The AIM2 inflammasome is also essential for host defense against bacterial and viral pathogens, such as *Francisella tularensis*, vaccinia virus, and mouse cytomegalovirus (Rathinam et al., 2010). In addition, some studies have identified important CNS roles for AIM2 such as anti-bacterial infection, brain injury, and shaping neurodevelopment (Denes et al., 2015; Hanamsagar et al., 2014; Lammert et al., 2020). However, the role of AIM2 in EAE and MS remains poorly understood.

Here, we report a previously unknown and inflammasome-independent protective role for AIM2 in EAE. Microglial AIM2 prevents the development of EAE by negatively regulating antiviral inflammatory signaling during neuroinflammation.

## Results

### AIM2 deficiency facilitates the development of both types A and B EAE

**Figure 1.**
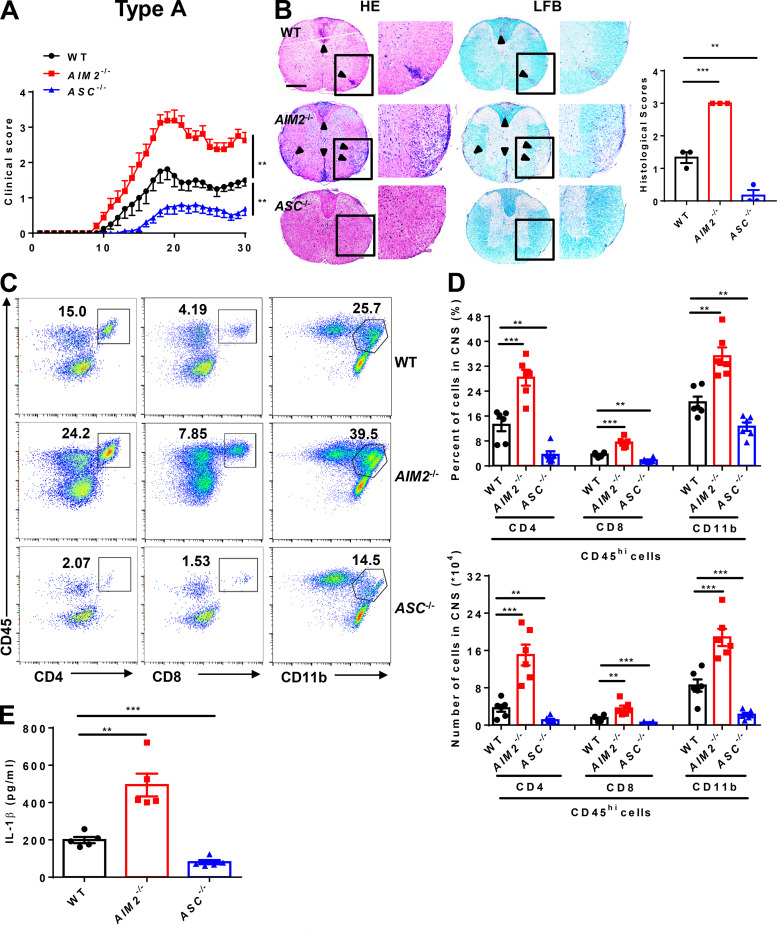
**AIM2 inhibits type A EAE development. (A)** Mean clinical scores of age-matched female WT, *AIM2*^−/−^, and *ASC*^−/−^ mice subjected to type A EAE (*n* = 8 mice per group). **(B)** Representative H&E and LFB staining and histology scores of spinal cord sections from type A EAE–induced WT, *AIM2*^−/−^, and *ASC*^−/−^ mice, showing inflammatory cell infiltration and demyelination (arrowheads). Scale bar, 500 µm. **(C and D)** Flow-cytometric analysis of immune cells (including CD45^+^CD4^+^ T cells, CD45^+^CD8^+^ T cells, and CD45^+^CD11b^+^ microglia and myeloid cells) infiltrated to the spinal cord and brain of type A EAE–induced WT, *AIM2*^−/−^, and *ASC*^−/−^ mice at day 18 after immunization (*n* = 6 mice per group). Data are presented as representative plots (C) and summary graphs of quantified percentages and absolute cell numbers (D). **(E)** ELISA analysis of IL-1β production in serum from indicated mice in type A EAE (*n* = 5 mice per group). Data are pooled from three independent experiments. **, P < 0.01; ***, P < 0.001. Error bars show mean ± SEM. Unpaired *t* test for A and E, and multiple unpaired *t* test for D.

**Figure 2.**
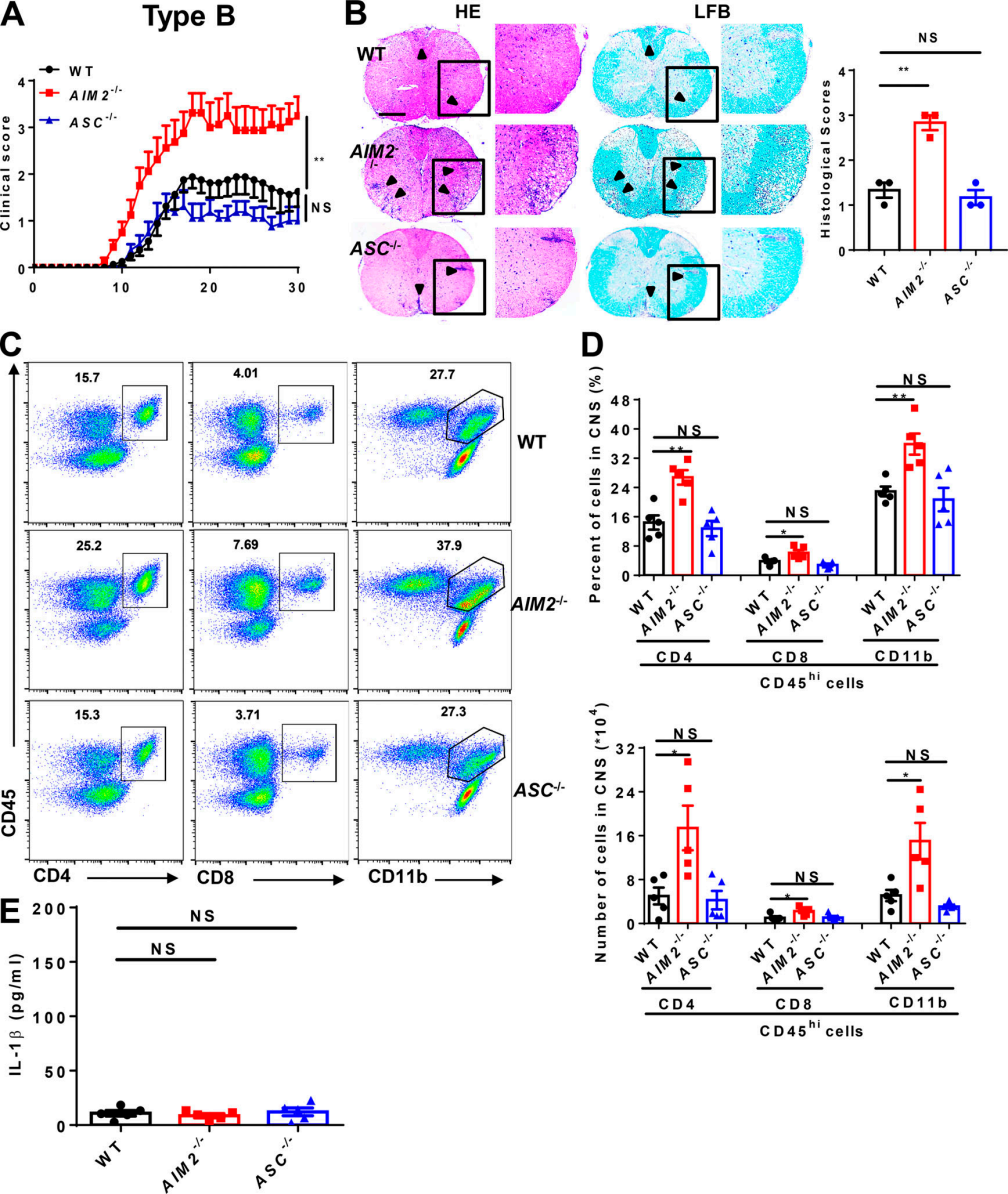
**AIM2 inhibits type B EAE development. (A)** Mean clinical scores of age-matched female WT, *AIM2*^−/−^, and *ASC*^−/−^ mice subjected to type B EAE (*n* = 8 mice per group). **(B)** Representative H&E and LFB staining and histology scores of spinal cord sections from type B EAE–induced WT, *AIM2*^−/−^, and *ASC*^−/−^ mice, showing inflammatory cell infiltration and demyelination (arrowheads). Scale bar, 500 µm. **(C and D)** Flow-cytometric analysis of immune cells (including CD45^+^CD4^+^ T cells, CD45^+^CD8^+^ T cells, and CD45^+^CD11b^+^ microglia and myeloid cells) infiltrated to the spinal cord and brain of type B EAE–induced WT, *AIM2*^−/−^, and *ASC*^−/−^ mice at day 18 after immunization (*n* = 5 mice per group). Data are presented as representative plots (C) and summary graphs of quantified percentages and absolute cell numbers (D). **(E)** ELISA analysis of IL-1β production in serum from indicated mice in type B EAE (*n* = 5 mice per group). Data are pooled from three independent experiments. *, P < 0.05; **, P < 0.01. Error bars show mean ± SEM. Unpaired *t* test for A and E, and multiple unpaired *t* test for D.

MOG-induced EAE has become a very well characterized model for investigating key components of the immune system in the pathogenesis of MS (Constantinescu et al., 2011) and has been separated into A and B subtypes to explore the heterogeneity in MS (Inoue et al., 2016). To more comprehensively elucidate the role of AIM2 in heterogeneous MS, we induced both type A and B EAE in WT and *AIM2*^−/−^ mice using different doses of Mtb. *ASC*^−/−^ mice were used as inflammasome-dependent type A EAE controls. Clinical scores and the levels of infiltrating inflammatory cells and demyelination in the spinal cord of *ASC*^−/−^ mice were lower in type A EAE (Fig. 1, A and B) but comparable in type B EAE relative to WT mice (Fig. 2, A and B), which is consistent with previous reports showing ASC as an essential mediator of type A EAE but not type B EAE. Interestingly, we found *AIM2*^−/−^ mice displayed higher clinical scores compared with WT mice in both types of EAE. In addition, CNS pathology was also elevated in *AIM2*^−/−^ mice during both types of EAE (Fig. 1, A and B; and Fig. 2, A and B). These data suggest that unlike other inflammasome proteins, AIM2 plays a protective role in the development of different types of EAE. Consistently, FACS analysis revealed a significantly increase in the infiltration of T cells (CD45^+^CD4^+^ and CD45^+^CD8^+^), myeloid cells, and activated microglia cells (CD45^high^CD11b^+^) in the CNS of *AIM2*^−/−^ mice during both types of EAE when compared with WT or *ASC*^−/−^ mice (Fig. 1, C and D; and Fig. 2, C and D). We also measured the release of mature IL-1β in serum during EAE. Low comparable levels of IL-1β were detected in WT, *ASC*^−/−^, and *AIM2*^−/−^ mice during type B EAE (Fig. 2 E). However, in type A EAE, *AIM2*^−/−^ mice showed higher levels of IL-1β in serum, but the levels of IL-1β in *ASC*^−/−^ mice were lower compared with WT mice (Fig. 1 E). Taken together, these results indicated that AIM2 controls the development of both types of EAE in an inflammasome-independent manner. To better explore the underlying inflammasome-independent mechanism by which AIM2 controls neuroinflammation during EAE, we used the type B EAE model in our subsequent experiments.

### AIM2 deficiency in microglia exacerbates neuroinflammation and the pathogenesis of EAE

**Figure 3.**
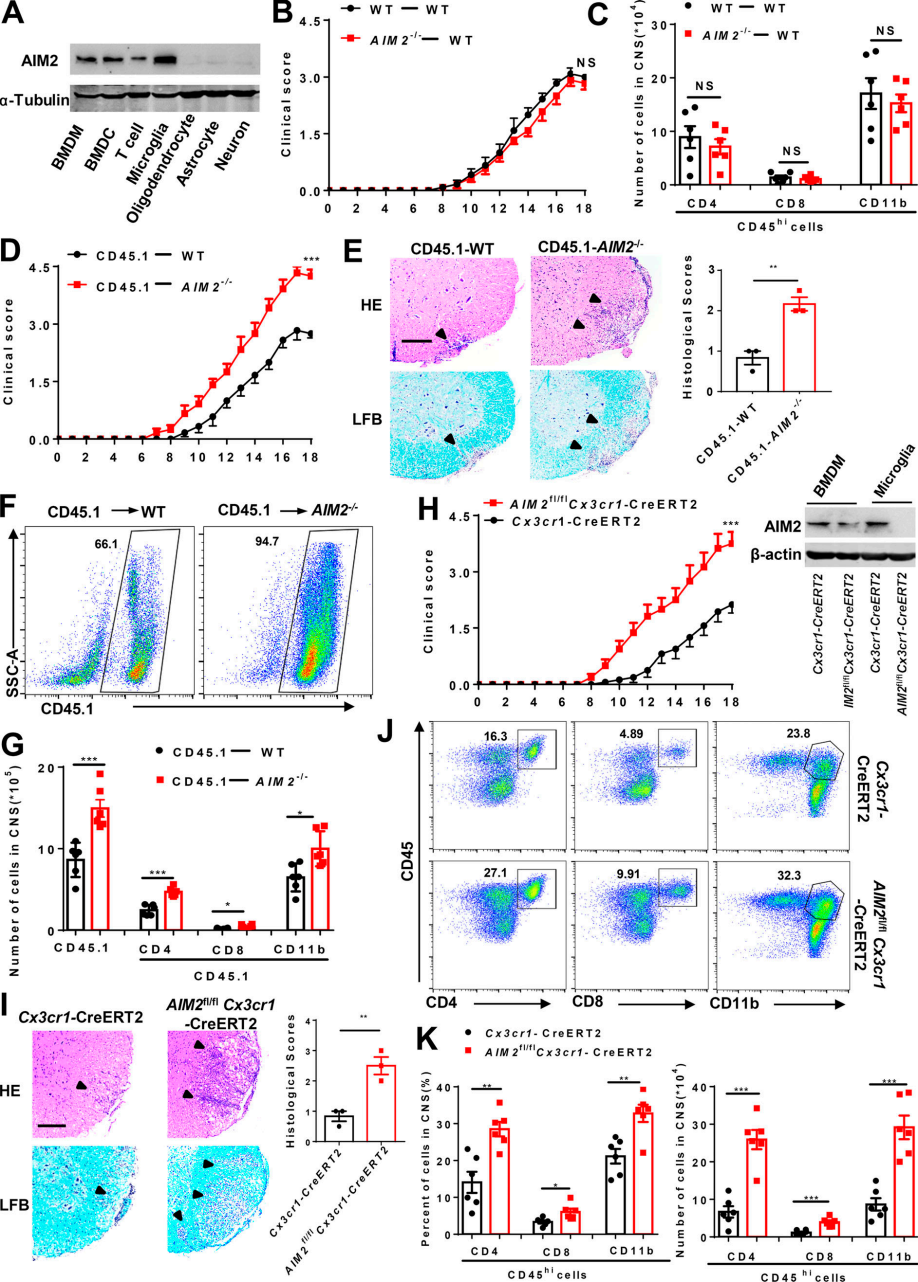
**AIM2 deletion in microglia promotes the development of EAE. (A)** Immunoblot analysis of the protein expression of AIM2 in peripheral cells (BMDMs, bone marrow–derived DCs [BMDCs], and T cells) and CNS resident cells (microglia, astrocytes, oligodendrocytes, and neurons) from WT mice. **(B and C)** Mean clinical scores (B) after EAE induction and summary graph of CNS-infiltrating immune cells (C) at day 18 after immunization in WT mice adoptively transferred with WT and *AIM2*^−/−^ bone marrow cells (*n* = 6 mice per group). **(D)** Mean clinical scores after EAE induction in WT and *AIM2*^−/−^ mice adoptively transferred with CD45.1 WT bone marrow cells (*n* = 6 mice per group). **(E)** Representative H&E and LFB staining and histology score of spinal cord sections harvested from the mice in D, showing inflammatory cell infiltration and demyelination (arrowheads). Scale bar, 200 µm. **(F and G)** Representative plot of CD45.1^+^ cells (F) and summary graph of CNS-infiltrating immune cells from FACS analysis of the mice in D (*n* = 6 mice per group; G). SSC-A, side scatter area. **(H)** Mean clinical scores of *AIM2*^fl/fl^*Cx3cr1*-CreERT2 and *Cx3cr1*-CreERT2 mice after EAE induction (*n* = 8 mice per group). Immunoblot analysis of AIM2 expression in microglia from the indicated mice. **(I)** Representative H&E and LFB staining and histology score of spinal cord sections harvested from the mice in H, showing inflammatory cell infiltration and demyelination (arrowheads). Scale bar, 200 µm. **(J and K)** FACS analysis of CNS-infiltrating immune cells (*n* = 6 mice per group) from the mice in H. Data in are presented as a representative plot (J), quantified percentage, and absolute cell numbers (K). Data are pooled from three independent experiments. *, P < 0.05; **, P < 0.01; ***, P < 0.001. Error bars show mean ± SEM. Unpaired *t* test for B, D, and H, and multiple unpaired *t* test for C, G, and K.

Next, we measured AIM2 protein expression in a variety of immune and nerve cells. Immunoblotting analysis showed that AIM2 was highly expressed in bone marrow–derived macrophages (BMDMs), bone marrow–derived dendritic cells (DCs), and microglia. AIM2 was expressed at a lower level in T cells, and was barely detectable in astrocytes, oligodendrocytes, and neurons (Fig. 3 A). To further determine whether AIM2 deficiency in CNS resident cells or peripheral immune cells contributes to the regulation of EAE, we performed bone marrow chimera experiments by adoptively transferring WT or *AIM2*^−/−^ bone marrow cells into lethally irradiated WT recipient mice to determine the function of AIM2 in peripheral cells on EAE. Comparable clinical scores (Fig. 3 B) and infiltration of immune cells (Fig. 3 C) were observed between these two recipients, indicating that AIM2 in peripheral cells is not involved in protecting against EAE. We next performed a reverse bone marrow transfer experiment by reconstituting lethally irradiated WT or *AIM2*^−/−^ mice with CD45.1 WT bone marrow cells. We found that *AIM2*^−/−^ mice reconstituted with CD45.1 WT bone marrow showed higher clinical scores (Fig. 3 D) and more CNS infiltration of immune cells (Fig. 3, F and G). H&E and Luxol fast blue (LFB) staining also showed more inflammatory cell recruitment and increased demyelination in the spinal cord of *AIM2*^−/−^ recipient mice (Fig. 3 E). Thus, these results suggested that AIM2 deficiency in CNS-resident cells promotes the pathogenesis of EAE.

**Figure S1.**
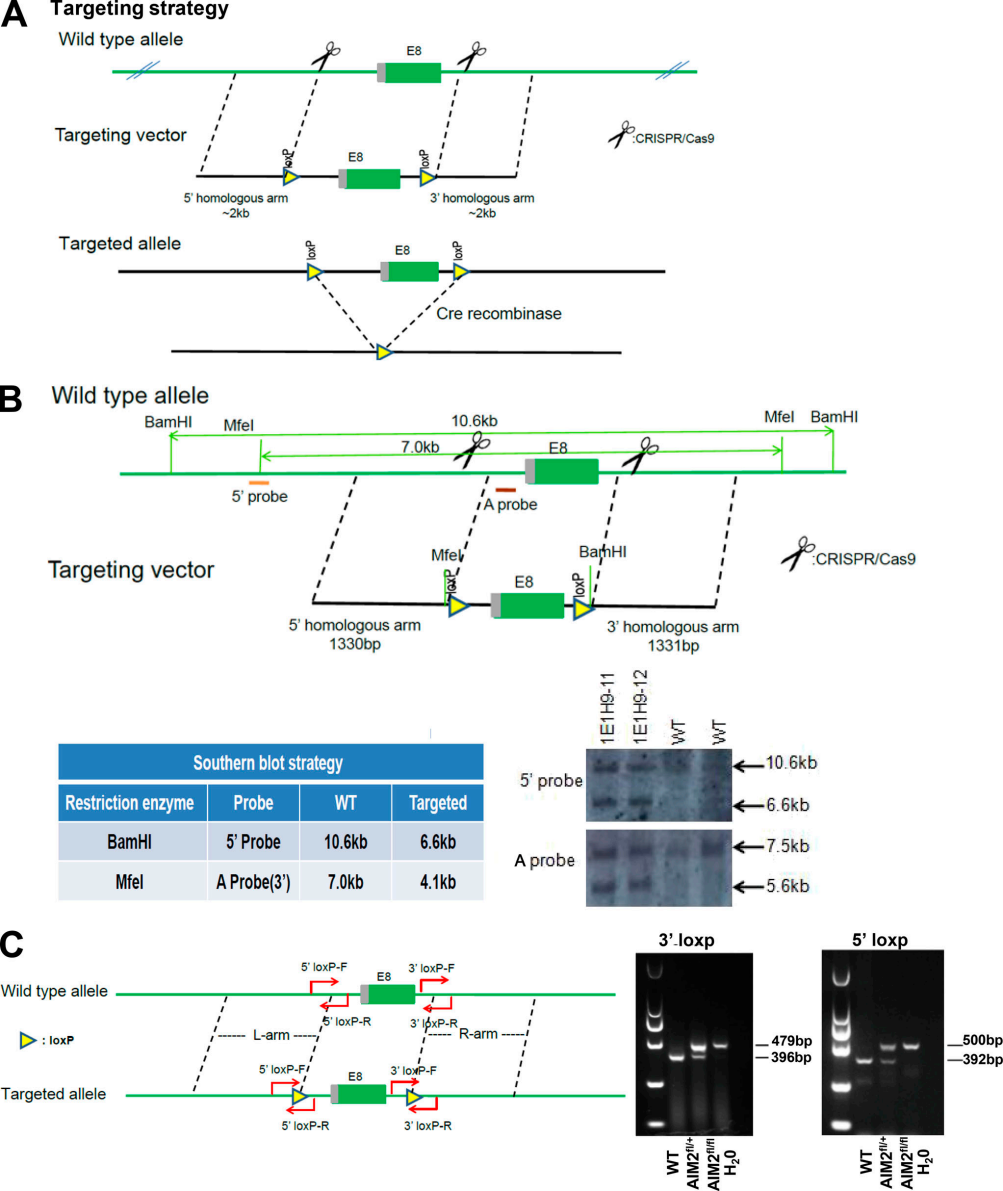
**AIM2 conditional KO mice strategy.** **(A)** Targeting vector design for generation of a mouse strain with AIM2 exon 5 flanked by LoxP sites. **(B)** Regions selected for Southern blotting and the result of Southern blotting analysis. **(C)** Genotyping strategy and representative image for genotyping analysis of AIM2 LoxP sites.

**Figure S2.**
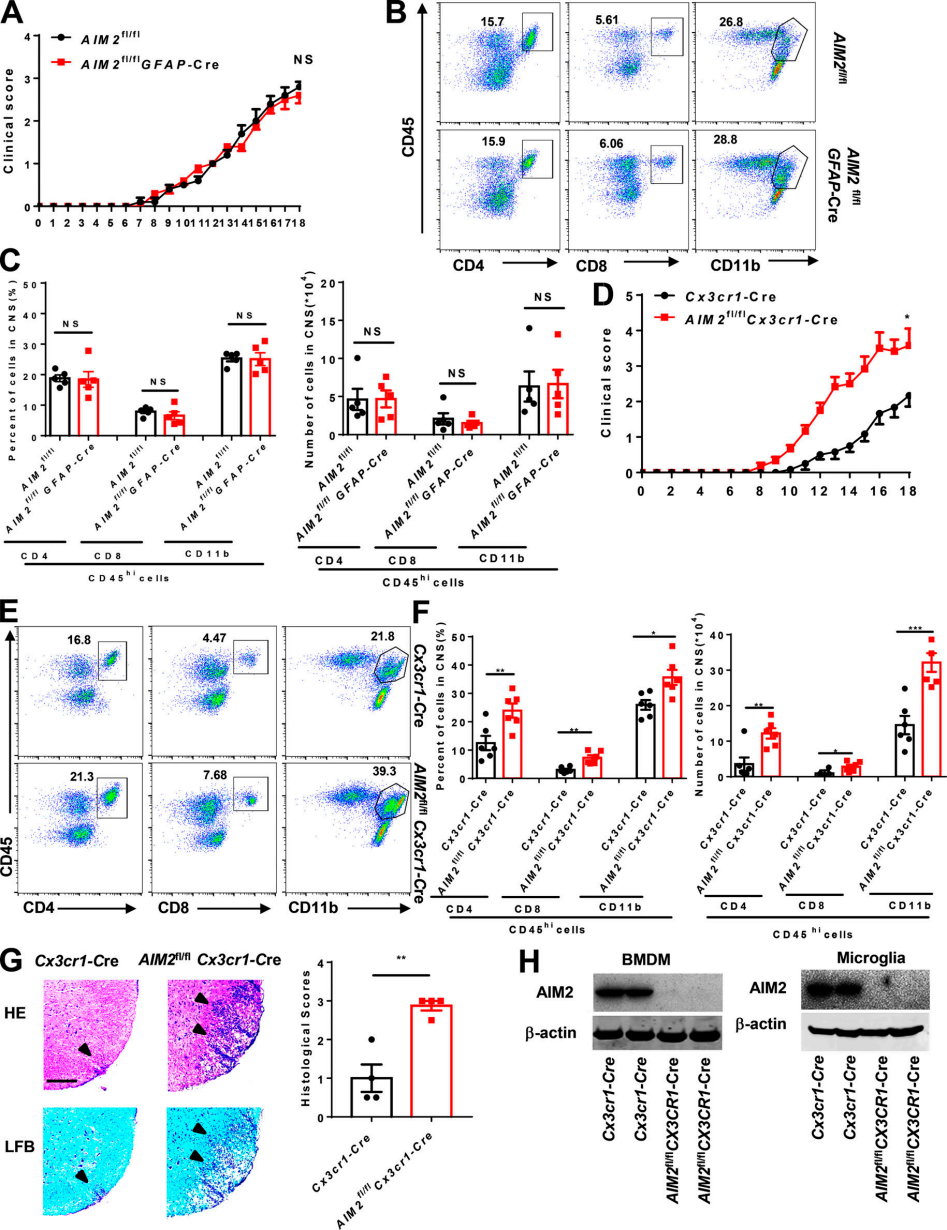
**AIM2 deletion in microglia but not in astrocyte promotes the development of EAE. (A)** Mean clinical scores after EAE induction in *AIM2*^fl/fl^ and *AIM2*^fl/fl^*GFAP*-Cre mice (*n* = 5 mice per group). **(B and C)** FACS analysis of immune cells infiltrated to the CNS of the mice in A at day 18 after immunization (*n* = 5 mice per group). Data are presented as a representative plot (B) and quantified percentage and absolute cell numbers (C). **(D)** Mean clinical scores after EAE induction in *Cx3cr1*-Cre and *AIM2*^fl/fl^*Cx3cr1*-Cre mice (*n* = 6 mice per group). **(E and F)** FACS analysis of immune cells infiltrated to the CNS of the mice in D at day 18 after immunization (*n* = 6 mice per group). Data are presented as a representative plot (E), quantified percentage, and absolute cell numbers (F). **(G)** Representative H&E and LFB staining and histology score of spinal cord sections from EAE-induced *Cx3cr1*-Cre and *AIM2*^fl/fl^*Cx3cr1*-Cre mice, showing inflammatory cell infiltration and demyelination (arrowheads). Scale bar, 200 µm. **(H)** Immunoblot analysis of AIM2 expression in BMDMs and microglia from indicated mice. Data are pooled from three independent experiments. *, P < 0.05; **, P < 0.01; ***, P < 0.001. Error bars show mean ± SEM. Unpaired *t* test for A and D, and multiple unpaired *t* test for C and F.

**Figure S3.**
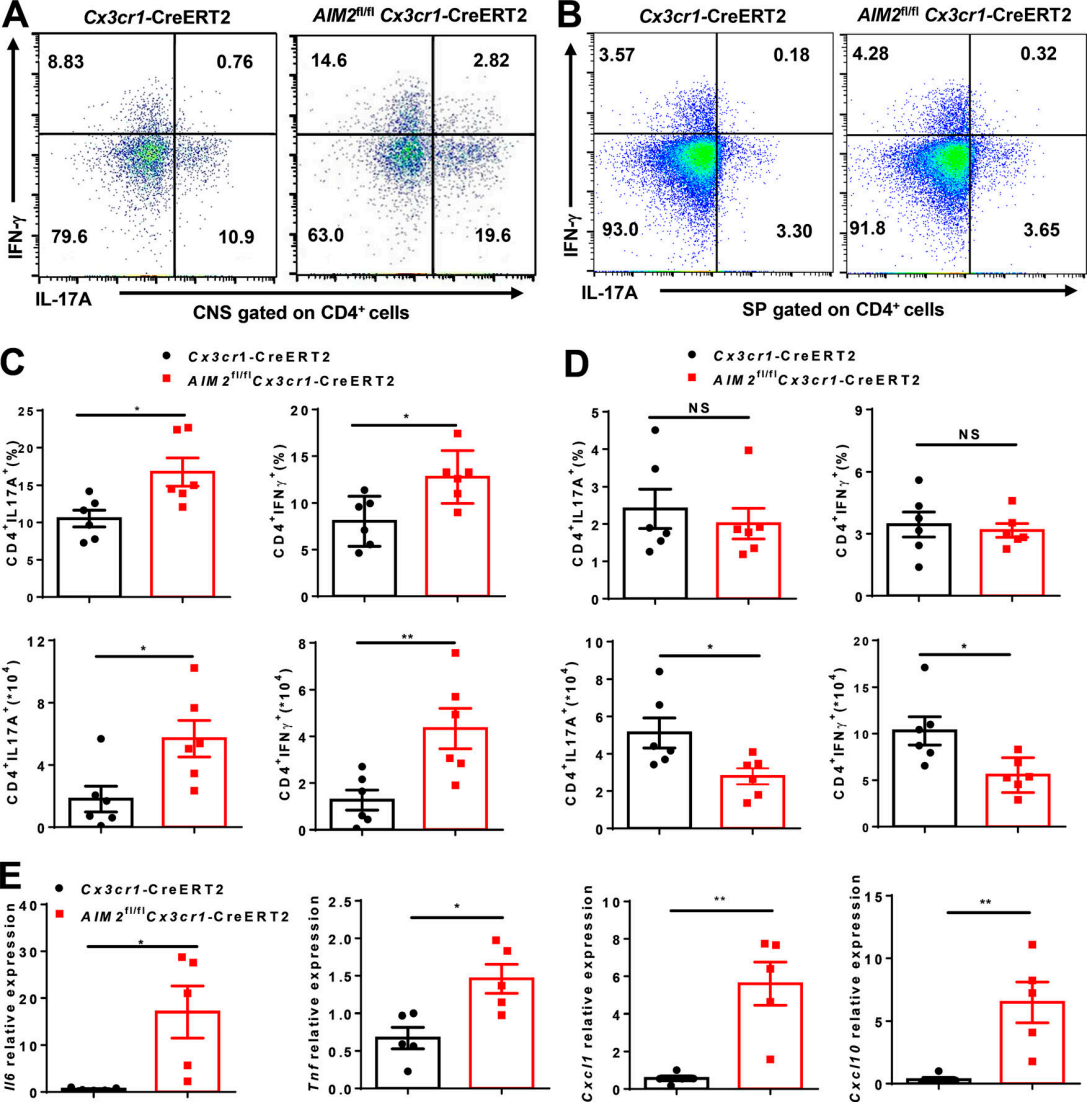
**AIM2 deletion in microglia promotes the development of EAE. (A and C)** FACS analysis of Th1 (IFN-γ^+^) and Th17 (IL-17A^+^) cells from CD4^+^ T cells infiltrated to the CNS of type B EAE-induced *AIM2*^fl/fl^*Cx3cr1*-CreERT2 and *Cx3cr1*-CreERT2 mice at day 18 after immunization (*n* = 6 mice per group). Data are presented as a representative plot (A), quantified percentage, and absolute cell numbers (C). **(B and D)** FACS analysis of Th1 (IFN-γ^+^) and Th17 (IL-17A^+^) cells from CD4^+^ T cells infiltrated to the spleens of type B EAE-induced *AIM2*^fl/fl^*Cx3cr1*-CreERT2 and *Cx3cr1*-CreERT2 mice at day 18 after immunization (*n* = 6 mice per group). Data are presented as a representative plot (B), quantified percentage, and absolute cell numbers (D). SP, spleen. **(E)** Quantitative PCR analysis of the relative mRNA expression of proinflammatory cytokines and chemokines in the spinal cord of indicated mice (*n* = 5 mice per group) at the EAE peak. Data were normalized to a reference gene, *Hprt*. Data are pooled from three independent experiments. *, P < 0.05; **, P < 0.01. Error bars show mean ± SEM. Unpaired *t* test.

We also crossed *AIM2*^fl/fl^ mice with *GFAP*-Cre mice to generate astrocyte conditional AIM2 KO mice (Fig. S1, A–C). During EAE, *AIM2*^fl/fl^*GFAP*-Cre and littermate control *AIM2*^fl/fl^ mice had comparable susceptibility to EAE (Fig. S2, A–C). Consistent with low expression of AIM2 in astrocytes, these results demonstrate that AIM2 deficiency in astrocytes has no effect on EAE. To determine the role of AIM2 in microglia, we crossed *AIM2*^fl/fl^ mice with *Cx3cr1*-Cre mice to generate microglia and macrophage conditional AIM2 KO mice. Clinical scores and immune cell infiltration were significantly increased in *AIM2*^fl/fl^*Cx3cr1*-Cre mice compared with *Cx3cr1*-Cre mice (Fig. S2, D–H). Given *AIM2*^fl/fl^*Cx3cr1*-Cre mice deleted both microglia and peripheral macrophages, we crossed *AIM2*^fl/fl^ mice with *Cx3cr1*-CreERT2 mice to further generate microglia-specific conditional AIM2 KO mice. Tamoxifen was administered to these mice to specifically delete AIM2 in microglia. Short-lived blood monocytes can be renewed within 6 wk after administration of tamoxifen. However, the long-lived microglia are unable to regenerate. *AIM2*^fl/fl^*Cx3cr1*-CreERT2 mice developed more severe EAE with higher clinical scores, increased pathological features, and more immune cell infiltration when compared with *Cx3cr1*-CreERT2 mice (Fig. 3, H–K). Furthermore, the percentages and absolute numbers of both T helper type 1 (Th1) and Th17 cells were also significantly increased in the CNS of *AIM2*^fl/fl^*Cx3cr1*-CreERT2 mice (Fig. S3, A and C). However, the absolute numbers of Th1 and Th17 cells in the spleen of *AIM2*^fl/fl^*Cx3cr1*-CreERT2 mice were decreased, while percentages were comparable to control mice (Fig. S3, B and D). Thus, these results suggest that the recruitment of pathogenic T cells into the CNS is substantially enhanced in *AIM2*^fl/fl^*Cx3cr1*-CreERT2 EAE mice. Consistently, the relative expression of inflammatory cytokines and chemokines in CNS of *AIM2*^fl/fl^*Cx3cr1*-CreERT2 mice was markedly increased relative to *Cx3cr1*-CreERT2 mice during EAE (Fig. S3 E). Collectively, these data demonstrated that the specific loss of AIM2 in microglia is sufficient to exacerbate type B EAE.

### AIM2 controls the microglial phenotypic switch from homeostasis to disease during EAE

**Figure 4.**
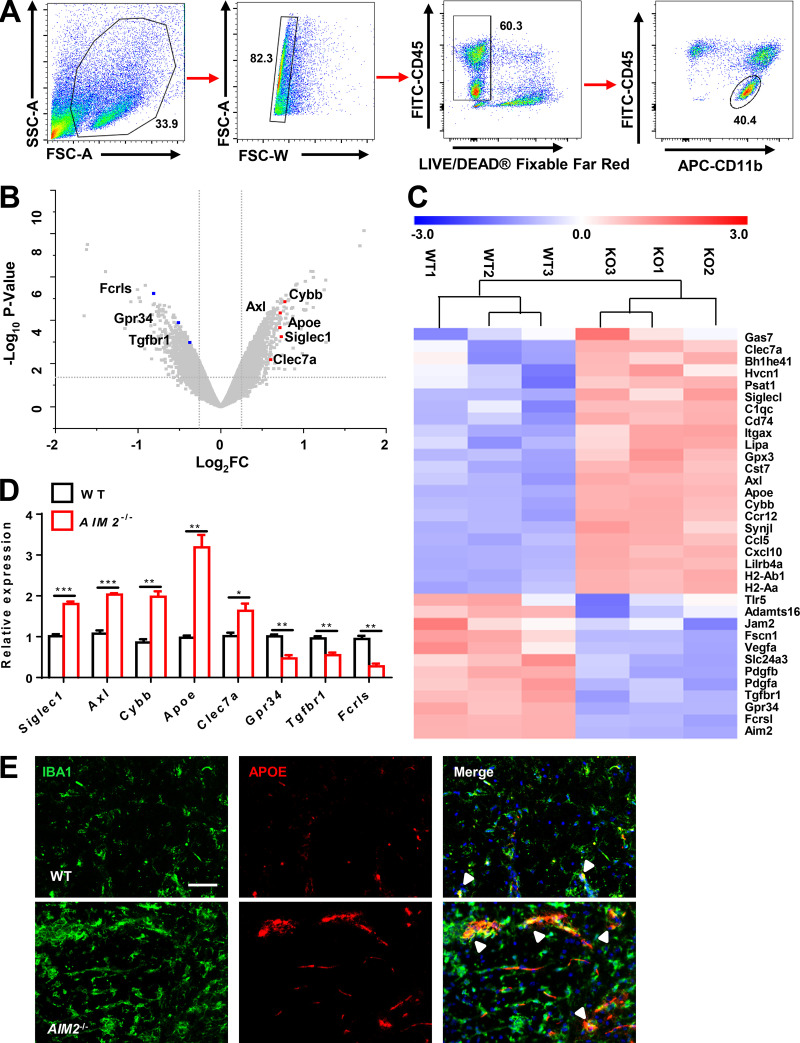
**AIM2 deficiency drives the microglial disease phenotypic switch during EAE. (A)** FACS sorting strategy of CD45^low^CD11b^+^ microglia cells from CNS in WT and *AIM2*^−/−^ mice at day 18 after immunization. SSC-A, side scatter area; FSC-A, forward scatter area; FSC-W, forward scatter pulse width. **(B)** Volcano plot displaying the results of differential gene expression analysis performed in CD45^low^CD11b^+^ microglia cells isolated from the CNS of WT and *AIM2*^−/−^ mice at day 18 after immunization. Genes related with disease-associated microglia signature and M0 signature are indicated. **(C)** The heatmap of genes with adjusted P value <0.05, false discovery rate <0.05, and log2 fold-change (FC) >1.2 from microarray analysis of CD45^low^CD11b^+^ microglia cells isolated from the CNS of WT and *AIM2*^−/−^ mice at day 18 after immunization. **(D)** Quantitative PCR analysis of the expression of indicated genes in CD45^low^CD11b^+^ microglia cells sorted from CNS in C. Data were normalized to a reference gene, *Hprt*. **(E)** Immunofluorescence analysis APOE expression in the microglia (IBA1^+^) of spinal cords from *AIM2*^−/−^ and WT mice during EAE. The merge of APOE with IBA1 is indicated by arrowheads. Scale bar, 50 µm. Data are representative of three independent experiments (E). Error bars show mean ± SEM. *, P < 0.05; **, P < 0.01; ***, P < 0.001. Multiple unpaired *t* test.

It is clear from the above results that microglia-intrinsic AIM2 regulates the development of EAE. To further dissect the mechanistic role of microglial AIM2 in controlling EAE, we performed microarray analysis using CD45^low^CD11b^+^ microglial cells sorted from the CNS of WT and *AIM2*^−/−^ mice on day 18 after EAE immunization (Fig. 4 A). Interestingly, volcano plot analysis displayed significantly increased expression in many genes related to a disease-associated microglia signature, such as *Apoe*, *Axl*, *Siglec1*, *Cybb*, and *Clec7a*, in *AIM2*^−/−^ cells, whereas many genes associated with a homeostatic microglia (M0) signature, such as *Fcrls*, *Gpr34*, and *Tgfbr1*, were down-regulated (Fig. 4 B). The heatmap and real-time PCR analysis confirmed these changes (Fig. 4, C and D). Consistently, immunofluorescence analysis showed that APOE expression was increased in microglia of *AIM2*^−/−^ mice compared with WT mice during EAE (Fig. 4 E). Together, these data suggest that AIM2 plays an important role in controlling the phenotypic switch of homeostatic microglia to inflammatory microglia during EAE.

### AIM2 deficiency enhances the microglial antiviral pathway–related inflammation during EAE

**Figure 5.**
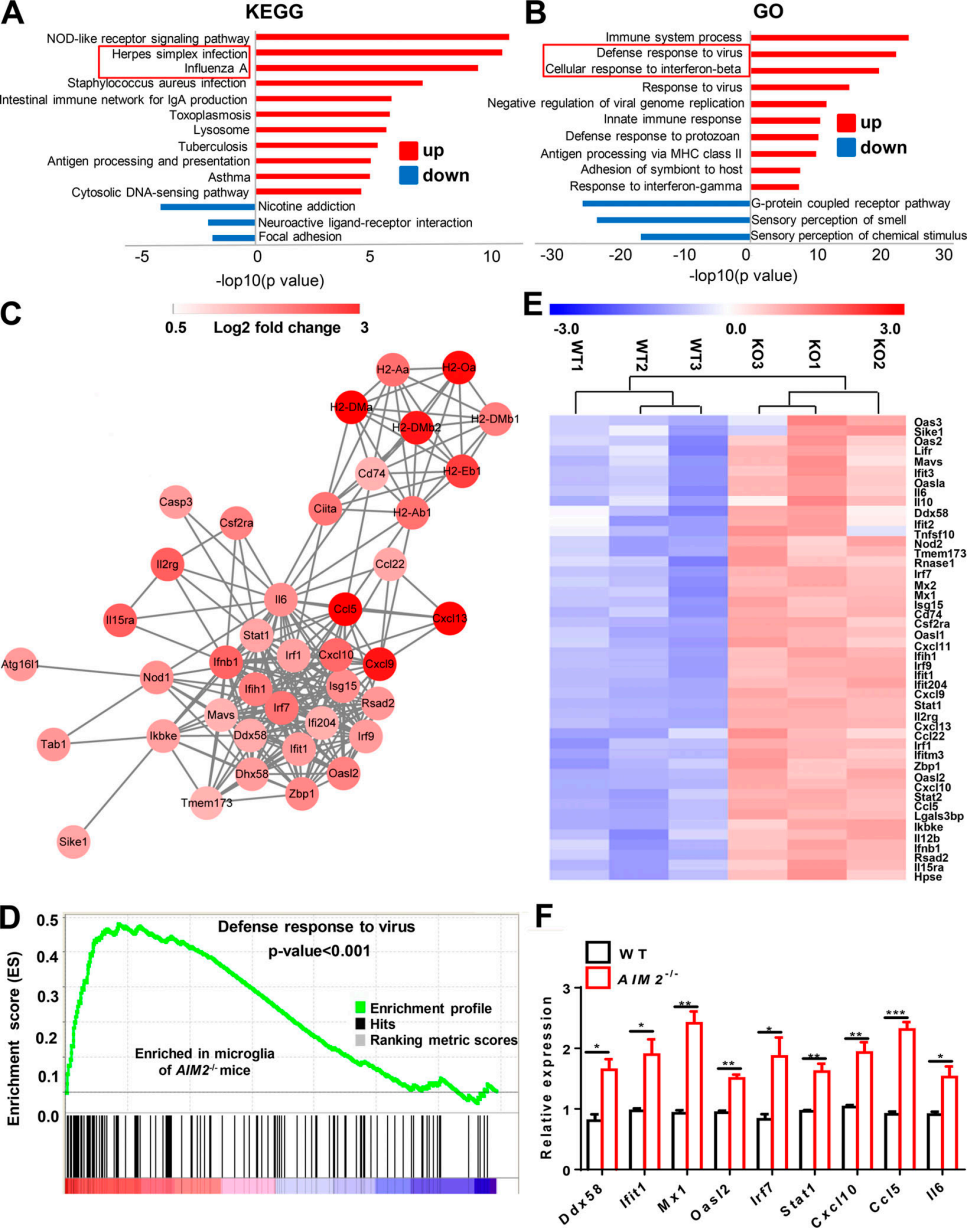
**AIM2 deficiency enhances the antiviral pathway–related inflammation of microglia during EAE. (A and B)** KEGG pathway analysis (A) and GO analysis (B) show the most significantly enriched signaling pathways in CD45^low^CD11b^+^ microglia cells sorted from the CNS of WT and *AIM2*^−/−^ mice at day 18 after immunization. **(C)** Gene network analysis of the genes associated with antiviral signaling pathway in microglia sorted from mice in A. Node colors from white to red indicate the changes of expression-level in AI*M2^−/−^* cells relative to WTs. **(D)** GSEA for the genes associated with “defense response to virus” in microglia sorted from mice in A using the Gene Ontology Biological Process Database. Nominal P < 0.001. **(E)** The heatmap of genes with adjusted P value <0.05, false discovery rate <0.05, and log2 fold-change >1.2 from microarray analysis of CD45^low^CD11b^+^ microglia cells isolated from the CNS of three pairs of WT and *AIM2*^−/−^ mice at day 18 after immunization. **(F)** Quantitative PCR analysis of indicated genes in CD45^low^CD11b^+^ microglia cells sorted from CNS in C (*n* = 3 mice per group). Data were normalized to a reference gene, *Hprt*. Error bars show mean ± SEM. *, P < 0.05; **, P < 0.01; ***, P < 0.001. Multiple unpaired *t* test.

To further identify the pathways involved in AIM2 controlling microglial inflammation, we did in-depth analysis of the microarray data. Notably, Kyoto Encyclopedia of Genes and Genomes (KEGG) analysis showed the up-regulation of many viral-related pathways in *AIM2*^−/−^CD45^low^CD11b^+^ cells, including herpes simplex infection, influenza A, and cytosolic DNA–sensing pathway (Fig. 5 A). Gene ontology biological process (GO) analysis further confirmed the top biological processes up-regulated in *AIM2*^−/−^ microglia were related with defense response to virus and cellular response to interferon-β (Fig. 5 B). Additionally, gene network and gene set enrichment analysis (GSEA) highlighted the regulated role of AIM2 in genes involved in the defense response to virus pathway (Fig. 5, C and D). Consistently, the heatmap and real-time PCR analysis displayed significantly increased expression of many genes associated with antiviral pathways and DNA-sensing pathways, such as *Ddx58*, *Ifit1*, *Mx1*, *Oasl2*, *Irf7*, *Stat1*, *Cxcl10*, *Ccl5*, and *Il6* in *AIM2*^−/−^ cells (Fig. 5, E and F). Thus, these genomic analyses clearly demonstrate that AIM2 restricts antiviral inflammatory signaling pathways in microglia.

### AIM2 targets the DNA-PK (DNA-dependent protein kinase)–AKT3 axis to inhibit the microglial antiviral pathway-related inflammation

**Figure 6.**
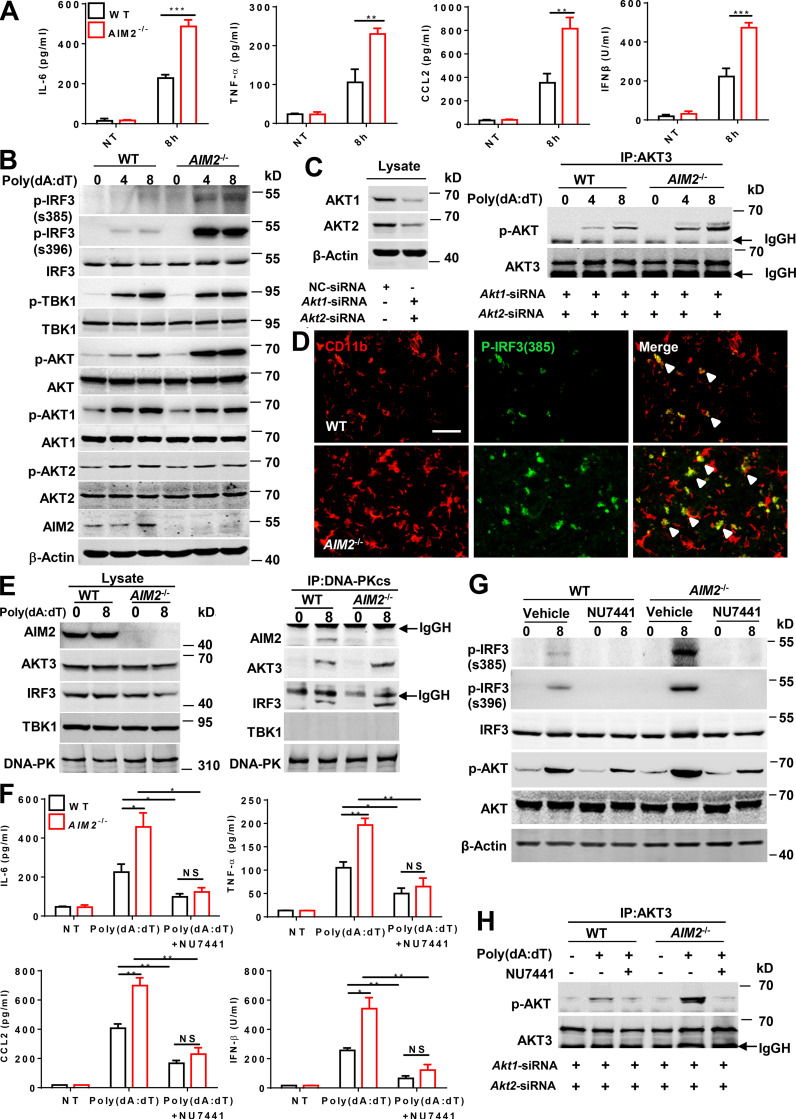
**AIM2 inhibits the microglial antiviral pathway–related inflammation by suppressing the DNA-PK–AKT3 signaling axis. (A)** ELISA analysis of TNF-α, IL-6, CCL2, and IFN-β in the supernatants of WT and *AIM2^−/−^* microglia transfected with poly(dA:dT) for 8 h. **(B)** Immunoblot analysis of p-IRF3(S385), p-IRF3(S396), IRF3, p-TBK1, TBK1, p-AKT, AKT, p-AKT1, AKT1, p-AKT2, AKT2, AIM2, and β-actin (loading control) in WT and *AIM2^−/−^* microglia transfected with poly(dA:dT) for 4 and 8 h. **(C)** WT and *AIM2^−/−^* microglia were transfected with siRNAs to silence *Akt1* and *Akt2* for 48 h, and then were transfected with poly(dA:dT) for 4 and 8 h. Cell lysates were immunoprecipitated (IP) with anti-AKT3 antibody followed by immunoblotting with p-AKT and AKT3. **(D)** Immunofluorescent labeling of p-IRF3(S385) (green) and CD11b (red) in the spinal cord of EAE-induced WT mice at day 18 after immunization. The merge of p-IRF3(S385) with CD11b is indicated by arrowheads. Scale bar, 50 µm. **(E)** WT and *AIM2^−/−^* microglia were transfected with poly(dA:dT) for 8 h. Then cell lysates were immunoprecipitated with anti-DNA-PK antibody followed by immunoblotting with DNA-PKcs, TBK1, IRF3, AKT3, and AIM2. **(F)** WT and *AIM2^−/−^* microglia were treated with DNA-PK inhibitor NU7441 (1 nM) and then were transfected with poly(dA:dT) for 8 h. The production of indicated cytokines and chemokines was analyzed by ELISA. **(G)** WT and *AIM2^−/−^* microglia were treated with DNA-PK inhibitor NU7441 (1 nM) and then were transfected with poly(dA:dT) for 8 h. The expression of p-IRF3(S385), p-IRF3(S396), IRF3, p-AKT, AKT, and β-actin (loading control) was analyzed by immunoblot. **(H)** WT and *AIM2^−/−^* microglia were transfected with siRNAs to silence Akt1 and Akt2 for 48 h, and then treated with DNA-PK inhibitor NU7441 (1 nM) followed by poly(dA:dT) transfection for 8 h. Cell lysates were immunoprecipitated with anti-AKT3 antibody followed by immunoblotting with p-AKT and AKT3. Data are pooled from three independent experiments for A and F. Data are representative of three (B and G) or two (C, E, and H) independent experiments. *, P < 0.05; **, P < 0.01; ***, P < 0.001. Error bars show mean ± SEM. Unpaired *t* test. NT, no treatment.

**Figure S4.**
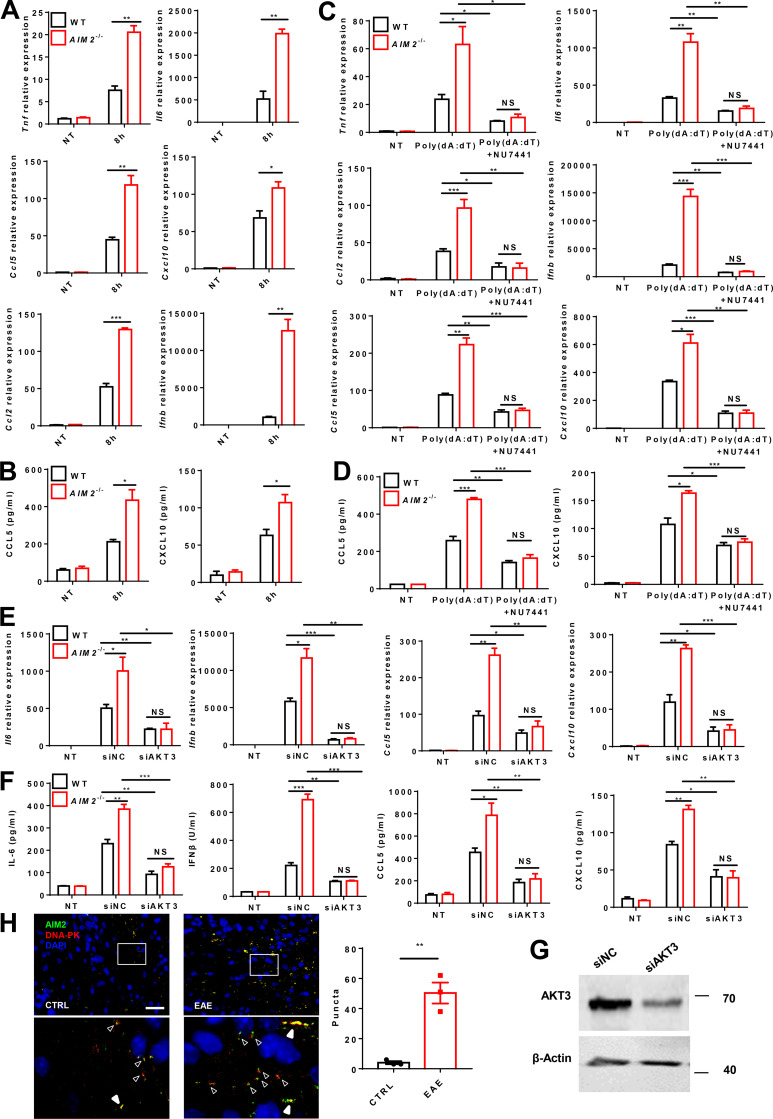
**AIM2 inhibits the microglial antiviral pathway-related inflammation by targeting DNA-PK. (A)** RT-qPCR analysis of *Ccl5* and *Cxcl10* mRNA expression of WT and *AIM2^−/−^* microglia transfected with poly(dA:dT) for 8 h. **(B)** ELISA analysis of CCL5 and CXCL10 in the supernatants of WT and *AIM2^−/−^* microglia transfected with poly(dA:dT) for 8 h. **(C)** WT and *AIM2^−/−^* microglia were treated with DNA-PK inhibitor NU7441 (1 nM) and then were transfected with poly(dA:dT) for 8 h. The mRNA expression of indicated cytokines and chemokines was analyzed by RT-qPCR. **(D)** WT and *AIM2^−/−^* microglia were treated with DNA-PK inhibitor NU7441 (1 nM) and then were transfected with poly(dA:dT) for 8 h. The production of indicated chemokines was analyzed by ELISA. **(E and F) **WT and *AIM2^−/−^* microglia were transfected with siRNAs to silence *Akt3* for 48 h, and then were transfected with poly(dA:dT) for 4 and 8 h. The mRNA expression (F) or protein levels (G) of indicated cytokines and chemokines were analyzed by RT-qPCR or ELISA. **(G)** Immunoblot analysis of AKT3 expression in WT microglia transfected with siRNAs to silence *Akt3* for 48 h. **(H)** Fluorescence in situ hybridization staining of *Aim2* and *Dnapk* mRNA in spinal cord from control and EAE-induced WT mice at day 18 after immunization. Quantification of Aim2 and Dnapk mRNA puncta colocalization (solid arrowheads) or in close proximity (hollow arrowheads) per 200× image (*n* = 3 mice per group). Scale bar, 100 µm. Data are pooled from three independent experiments. *, P < 0.05; **, P < 0.01; ***, P < 0.001. Error bars show mean ± SEM. Unpaired *t* test. NT, no treatment.

Poly(dA:dT) (poly(deoxyadenylic-deoxythymidylic)) transfection can strongly activate antiviral inflammatory pathways (Lees-Miller et al., 1990; Li et al., 2013), but barely do AIM2 inflammasome activation without first signal to prime (Hornung et al., 2009). We next investigated the inflammasome-independent effect of AIM2 in microglia on antiviral pathway–associated inflammation in response to poly(dA:dT) transfection. Indeed, we detected higher levels of antiviral-related cytokines and chemokines, such as IFN-β, CXCL10, CCL5, CCL2, IL-6, and TNF-α, in response to poly(dA:dT) in primary microglia from *AIM2*^−/−^ mice than those from WT mice (Fig. 6 A; and Fig. S4, A and B). AIM2 has been reported to restrict colon tumorigenesis by suppression of the DNA-PK–AKT axis in an inflammasome-independent manner (Wilson et al., 2015). Additionally, a recent study reported that AKT3 can phosphorylate IRF3 at Ser385, which facilitates TBK1-induced phosphorylation of IRF3 on Ser396 and enhances the antiviral inflammatory signaling (Xiao et al., 2020). We speculated that AIM2 in microglia may negatively regulate the DNA-PK–AKT3 axis to control antiviral pathway–related inflammation. To verify this hypothesis, we treated primary microglia from WT and *AIM2*^−/−^ mice with poly(dA:dT) and analyzed the phosphorylation of IRF3, TBK1, and AKTs. We observed an increase in the phosphorylation of IRF3 at Ser385, Ser396, and AKT, but not the phosphorylation of TBK1, AKT1, and AKT2 (Fig. 6 B) in *AIM2*^−/−^ microglia compared with controls after treatment. Since there is no commercial antibody to specifically detect p-AKT3, we silenced AKT1 and AKT2 in microglia, and immunoprecipitated AKT3 to detect AKT3 phosphorylation by using an anti-phosphorylated AKT antibody after poly(dA:dT) treatment. We found that AIM2 deficiency markedly enhanced poly(dA:dT)-induced AKT3 phosphorylation (Fig. 6 C), suggesting a negative role for AIM2 in regulating the activation of AKT3. Consistently, we also observed the enhanced phosphorylation of IRF3 at Ser385 in microglia from *AIM2*^−/−^ during EAE (Fig. 6 D). To further dissect the mechanism of AIM2 regulation of AKT3, we examined the interaction of the DNA-PK catalytic subunit (DNA-PKcs) with AIM2 and AKT3 in microglia following poly(dA:dT) treatment. Poly(dA:dT) induced the interaction of DNA-PKcs with AIM2, AKT3, and IRF3 but not with TBK1 in WT microglia. Interestingly, the interaction of DNA-PKcs with AKT3 and IRF3 was significantly increased in *AIM2*^−/−^ microglia (Fig. 6 E), suggesting that AIM2 suppresses recruitment of AKT3 and IRF3 to DNA-PK. Consistently, we observed great colocalization between AIM2 and DNA-PK in the spinal cord of EAE mice compared with untreated controls by using in situ hybridization (Fig. S4 H). To further verify that AIM2 targets the DNA-PK–AKT3 axis, the DNA-PK inhibitor NU7441 (Leahy et al., 2004) was used. WT and *AIM2*^−/−^ microglia were treated with NU7441 followed by poly(dA:dT) treatment. Treatment with NU7441 significantly reduced the elevated production of IFN-β, CXCL10, CCL5, CCL2, IL-6, and TNF-α in *AIM2^−^*^/−^ microglial cells (Fig. 6 F; and Fig. S4, C and D). In addition, NU7441 significantly reduced the phosphorylation of IRF3 at Ser385 and Ser396, and AKT3 in *AIM2*^−/−^ microglia to comparable levels in NU7441-treated WTs (Fig. 6, G and H). Moreover, we observed AKT3 knockdown significantly reduced the elevated production of IL-6, IFN-β, CXCL10, and CCL5 and in *AIM2^−^*^/^*^−^* microglial cells to comparable levels in AKT3-silenced WTs (Fig. S4, E–G). These data suggest AIM2 can inhibit the microglial antiviral inflammatory signaling by negatively regulating the recruitment of AKT3 and IRF3 to DNA-PK.

### Inhibition of the cGAS (cyclic guanosine monophosphate–adenosine monophosphate synthase)-DNA-PK axis rescues EAE pathogenesis in AIM2-deficient mice

**Figure S5.**
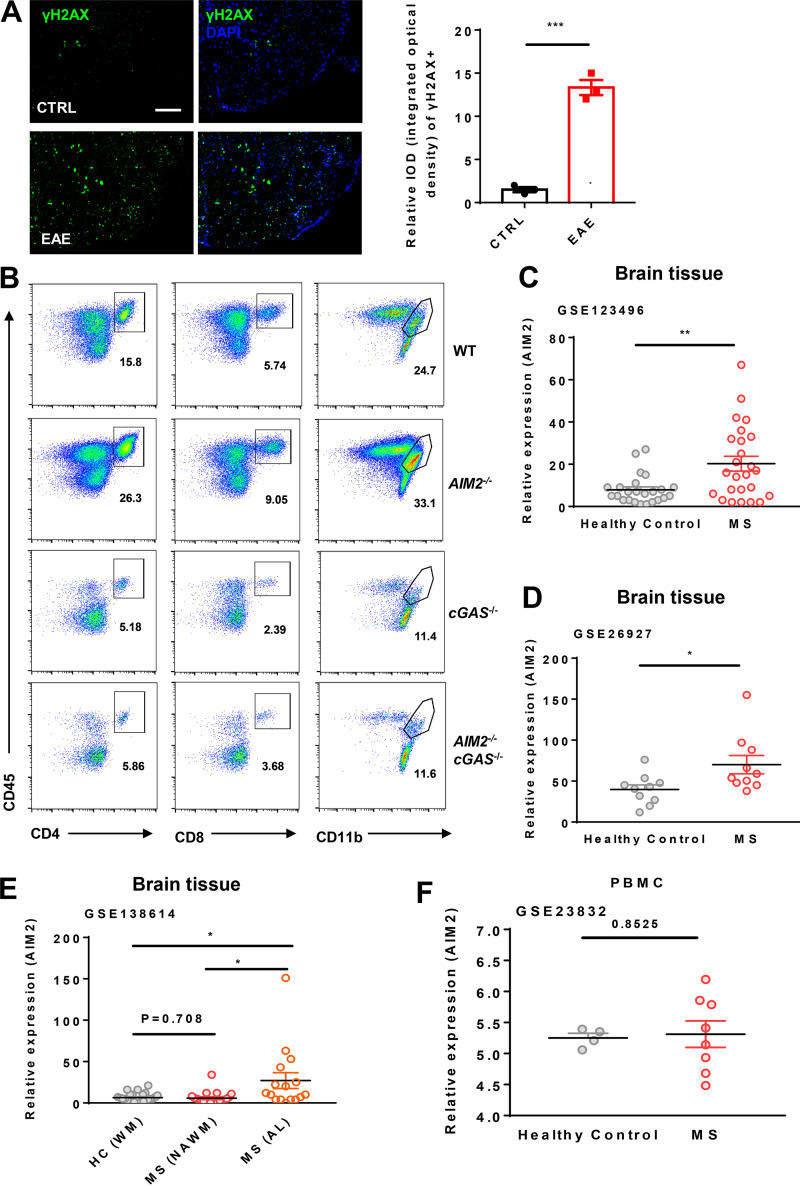
**AIM2 protects against cGAS-mediated neuroinflammation and AIM2 mRNA expression is increased in the brain tissues of MS patients. (A)** Spinal cord sections from control and EAE-induced WT mice at day 18 after immunization were evaluated for levels of DNA damage (γH2AX, green). Data are presented as representative images (left) and quantification of relative integrated optical density (IOD) of γH2AX (right; *n* = 3 mice per group). Scale bar, 200 µm. **(B)** Flow-cytometric analysis of immune cells (including CD45^+^CD4^+^ T cells, CD45^+^CD8^+^ T cells, and CD45^+^CD11b^+^ microglia and myeloid cells) infiltrated to the spinal cord and brain of EAE-induced WT, *AIM2*^−/−^, *cGAS*^−/−^, and *AIM2*^−/−^*cGAS*^−/−^ mice at day 18 after immunization (*n* = 5 mice per group). Data are presented as representative plots. **(C)** AIM2 mRNA expression in hippocampus, frontal cortex, internal capsule, corpus callosum, and parietal cortex of frozen autopsy samples from five female MS patients (average age, 57.6 yr) and five female age-matched healthy controls (average age, 57.2 yr); the data were from an RNA-seq database (accession no. GSE123496). **(D)** AIM2 mRNA expression in subpial gray matter lesions of the frontal gyri from 10 healthy (two female and eight male; average age at death, 53.1 yr) and 10 MS patients (five female and five male; average age at death, 49.4 yr), who were selected retrospectively on the basis of a confirmed clinical and neuropathological diagnosis, and snap-frozen brain blocks were provided by various tissue banks within the BrainNet Europe network; the data were from a microarray database (GEO accession no. GSE26927). **(E)** The AIM2 mRNA expression in a total of 25 white matter (WM) samples from five healthy controls without neurological disease, and 21 normal-appearing white matter (NAWM) and 16 active lesions from 10 progressive MS patients; the data were from a RNA-seq database (GEO accession no. GSE138614). AL, active lesions. **(F)** The AIM2 mRNA expression in peripheral blood mononuclear cells (PBMCs) from eight MS patients and four healthy controls (HC); the data were from microarray database (GEO accession no. GSE23832). *, P < 0.05; **, P < 0.01; ***, P < 0.001. Error bars show mean ± SEM. Unpaired *t* test. CTRL, control.

**Figure 7.**
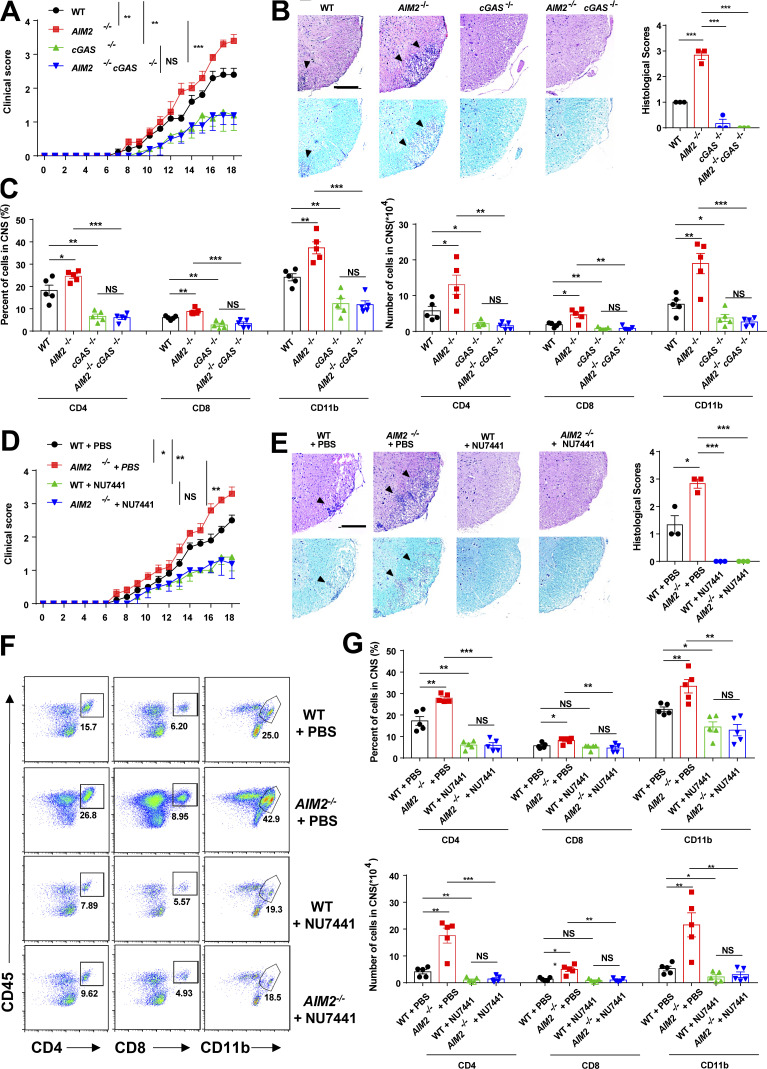
**Inhibition of cGAS-DNA-PK axis restricts EAE phenotype in AIM2^−/−^ mice. (A)** Mean clinical scores after EAE induction in WT, *AIM2*^−/−^, *cGAS*^−/−^, and *AIM2*^−/−^*cGAS*^−/−^ mice (*n* = 5 mice per group). **(B)** Representative H&E and LFB staining and histology score of spinal cord sections harvested from the mice in A, showing inflammatory cell infiltration and demyelination (arrowheads). Scale bar, 200 µm. **(C)** Flow-cytometric analysis of immune cells (including CD45^+^CD4^+^ T cells, CD45^+^CD8^+^ T cells, and CD45^+^CD11b^+^ microglia and myeloid cells) infiltrated to the spinal cord and brain of EAE-induced WT, *AIM2*^−/−^, *cGAS*^−/−^, and *AIM2*^−/−^*cGAS*^−/−^ mice at day 18 after immunization (*n* = 5 mice per group). Data are presented as summary graphs of quantified percentages and absolute cell numbers. **(D)** Mean clinical scores after EAE induction in WT and *AIM2*^−/−^ mice administrated with PBS or NU7441 at a dose of 10 mg/kg daily (*n* = 5 mice per group). **(E)** Representative H&E and LFB staining and histology score of spinal cord sections harvested from the mice in D, showing inflammatory cell infiltration and demyelination (arrowheads). Scale bar, 200 µm. **(F and G)** Flow-cytometric analysis of immune cells (including CD45^+^CD4^+^ T cells, CD45^+^CD8^+^ T cells, and CD45^+^CD11b^+^ microglia and myeloid cells) infiltrated to the spinal cord and brain of EAE-induced WT and *AIM2*^−/−^ mice administrated with PBS or NU7441 at a dose of 10 mg/kg daily at day 18 after immunization (*n* = 5 mice per group). Data are presented as representative plots (F) and summary graphs of quantified percentages and absolute cell numbers (G). Data are pooled from three independent experiments. *, P < 0.05; **, P < 0.01; ***, P < 0.001. Error bars show means ± SEM. Unpaired *t* test for A and D, and multiple unpaired *t* test for C and G.

Axonal damage and apoptotic loss of neurons occur in the brain during EAE (Hobom et al., 2004). Indeed, we observed a marked increase of DNA damage marker γH2AX in the spinal cord of mice during EAE (Fig. S5 A). DNA or cyclic dinucleotides released by dying nerve cells have been reported to activate cGAS-STING (stimulator of interferon genes) in microglia (Abdullah et al., 2018; Chin, 2019). To determine if AIM2 can restrict EAE by restraining cGAS-mediated inflammation, we crossed *AIM2*^−/−^ mice with *cGAS*^−/−^ mice to generate AIM2 and cGAS double-KO mice. During type B EAE, cGAS deficiency significantly alleviated clinical scores, inflammatory cell infiltration, and demyelination when compared with WT mice (Fig. 7, A–C). Furthermore, *AIM2*^−/−^*cGAS*^−/−^ were comparable to cGAS-deficient mice, as indicated by clinical score, inflammatory cell infiltration, and pathological features (Fig. 7, A–C; and Fig. S5 B). Thus, these data suggested that AIM2 plays a protective role in EAE by controlling cGAS-mediated EAE neuroinflammation. To further confirm that AIM2 controls EAE by inhibiting the DNA-PK–AKT3 axis, we administered WT and *AIM2*^−/−^ mice by i.p. injection with the DNA-PK inhibitor NU7441. Treatment of WT mice with NU7441 significantly attenuated the severity of EAE as indicated by lower clinical score, less inflammatory cell infiltration in CNS, and less demyelination (Fig. 7, D–G). Notably, NU7441 treatment also eliminated the augmentation of EAE severity in *AIM2*^−/−^ mice (Fig. 7, D–G). Overall, these data suggest that AIM2 protects against neuroinflammation by suppressing cGAS and DNA-PK–mediated inflammation.

## Discussion

Public RNA sequencing (RNA-seq) and microarray datasets in GEO (accession nos. GSE123496, GSE26927, and GSE138614) showed that AIM2 expression is increased in the brain tissues of MS patients when compared with healthy donors (Fig. S5, C–F), suggesting that AIM2 is induced during MS and may be involved in regulating the progression of MS (Durrenberger et al., 2015; Elkjaer et al., 2019; Voskuhl et al., 2019). Therefore, we used the EAE model to comprehensively dissect the role of AIM2 in the development of MS in this study.

Emerging evidence suggests the importance of inflammasome components of peripheral immune cells in the development of EAE by triggering T cell responses to initiate neuroinflammation (Inoue et al., 2012; Li et al., 2019b; Martin et al., 2016). However, in this study we found that AIM2 offers a protective function against EAE neuroinflammation via an unknown inflammasome-independent role, which differs from previous reports on the function of other inflammasome components in promoting EAE-induced neuroinflammation. Moreover, we found that AIM2 in microglia but not in peripheral cells is indispensable for its protective role in EAE, whereas other inflammasome proteins such as ASC and gasdermin D regulate EAE in T cells and peripheral myeloid cells (Li et al., 2019b; Martin et al., 2016). Although peripheral macrophages and DCs are involved in the onset of EAE, the amount of macrophages and DCs in the CNS tissue during EAE is much less than that of microglia (Bailey et al., 2007; Mrdjen et al., 2018). Moreover, microglia are the main CNS parenchyma-localized APCs required for infiltration and amplification of T cells during EAE (Archambault et al., 2005; Kawakami et al., 2004). Moreover, the public RNA-seq (GEO accession no. GSE52564) and single-cell RNA-seq (http://bis.zju.edu.cn/MCA/) data showed that AIM2 is highly expressed in microglia compared with other CNS cell types in mice, including neurons, astrocytes, and oligodendrocytes (Han et al., 2018; Zhang et al., 2014). Consistent with these published studies, our immunoblot analysis showed the expression of AIM2 in microglia was higher than in macrophages, DCs, astrocytes, and neurons. Thus, microglia-specific AIM2 plays a dominant role in modulating the pathogenesis of EAE. Although inflammasome proteins such as ASC, caspase-1, and AIM2 are enriched in microglial cells (Singhal et al., 2014), the AIM2 inflammasome requires a higher threshold of DNA for activation to occur (Choubey, 2019; Jones et al., 2010). Conversely, lower levels of DNA can be sensed directly by cGAS and DNA-PK (Ablasser and Chen, 2019; Choubey, 2019; Lees-Miller et al., 1990). The amount of DNA released by damaged neurons during EAE nerve injury may not be sufficient to stimulate AIM2-dependent inflammasome activation, which may explain why AIM2 in microglia functions in EAE independently of its inflammasome function.

Notably, we performed microarray analysis of microglia (CD45^low^CD11b^+^ cells) isolated from the CNS of WT and *AIM2*^−/−^ mice of EAE and revealed that the most prominent up-regulated responses in *AIM2*^−/−^ microglia from EAE-treated mice were associated with defense against viral infection. Correspondingly, a recent study showed an inflammasome-independent role of AIM2 in obesity and insulin resistance that is mediated by the up-regulation of Ifi202b and antiviral IFN signaling (Gong et al., 2019). Previous studies have implicated viral infections as triggers of neurodegenerative diseases such as Parkinson’s disease, Alzheimer’s disease, and amyotrophic lateral sclerosis (Amor et al., 2010; Deleidi and Isacson, 2012). Consistent with this, the antiviral cytokine IFN-β levels increase with age (Baruch et al., 2014; Yu et al., 2015). Additionally, viruses have been considered to be etiological agents of MS involved in demyelination (Libbey et al., 2014). Although IFN-β is the first-line treatment of relapsed and remitting MS at present, ∼50% of MS patients are nonresponsive to IFN-β. Furthermore, in some cases, IFN-β can exacerbate MS and consistently worsens neuromyelitis optica (Axtell et al., 2011; Río et al., 2006). Indeed, it has been reported that acute virus infection can induce the development of type B EAE in mice (Inoue et al., 2016). Significant increases in the mRNA levels of antiviral inflammatory genes such as *Ddx58*, *Ifit1*, *Oasl2*, *Irf7*, *Il6*, *Il12*, *Ccl22*, *Cxcl10*, and *Ccl5* were identified in microglia from *AIM2*^−/−^ EAE–treated mice. The increased expression of chemokines and cytokines such as *Cxcl10*, *Ccl22*, *Ccl5*, and *Il6* in *AIM2*^−/−^ cells plays a crucial role in T cell infiltration and cell proliferation during EAE (Balashov et al., 1999; Quandt and Dorovini-Zis, 2004; Takeda et al., 1998), which is critical step for the inflammation and pathogenesis of EAE or MS. Additionally, changes in the inflammatory state of microglia can affect its polarization and switching between M0 and microglial neurodegenerative phenotype (MGnD; Krasemann et al., 2017). We found the enhancement of MGnD genes (e.g., *Siglec1*, *Axl*, *Cybb*, *Clec7a*, and *Apoe*) and the suppression of M0 genes (e.g., *Siglec1*, *Gpr34*, *Tgfbr1*, and *Fcrsl*) in *AIM2*^−/−^ microglia during EAE. A previous study has suggested that MGnD microglia might play a detrimental role in EAE development (Krasemann et al., 2017). Moreover, the expression of genes involved in antigen processing and presentation, such as *H2-DMa*, *H2-DMb*, *H2-Aa*, *H2-Ab*, and *H2-Oa*, were also up-regulated in *AIM2*^−/−^ microglia of EAE. These MHC haplotypes have been established in EAE susceptibility (Iacobas et al., 2007; Welsh et al., 2016).

DNA-PK–associated proteins are expressed at the highest level in brain tissue (Moll et al., 1999), indicating an important role for the DNA-PK pathway in CNS. We revealed that AIM2 associates with DNA-PK in microglia and limits DNA-PK–mediated AKT3 recruitment and phosphorylation, thereby inhibiting downstream IRF3 activation and antiviral inflammatory signaling in neuroinflammation. The administration of a DNA-PK inhibitor dramatically attenuated the pathogenesis of EAE. Dying nerve cells have been reported to release DNA or cyclic dinucleotides to activate cGAS-STING–mediated inflammation in microglia (Abdullah et al., 2018). We found that the absence of cGAS precluded the augmented EAE pathogenesis that is observed in *AIM2*^−/−^ mice. Overall, our studies suggest that the development of therapeutic strategies to specifically target cGAS and DNA-PK signaling might be useful for the treatment of heterogeneous MS.

**Figure 8.**
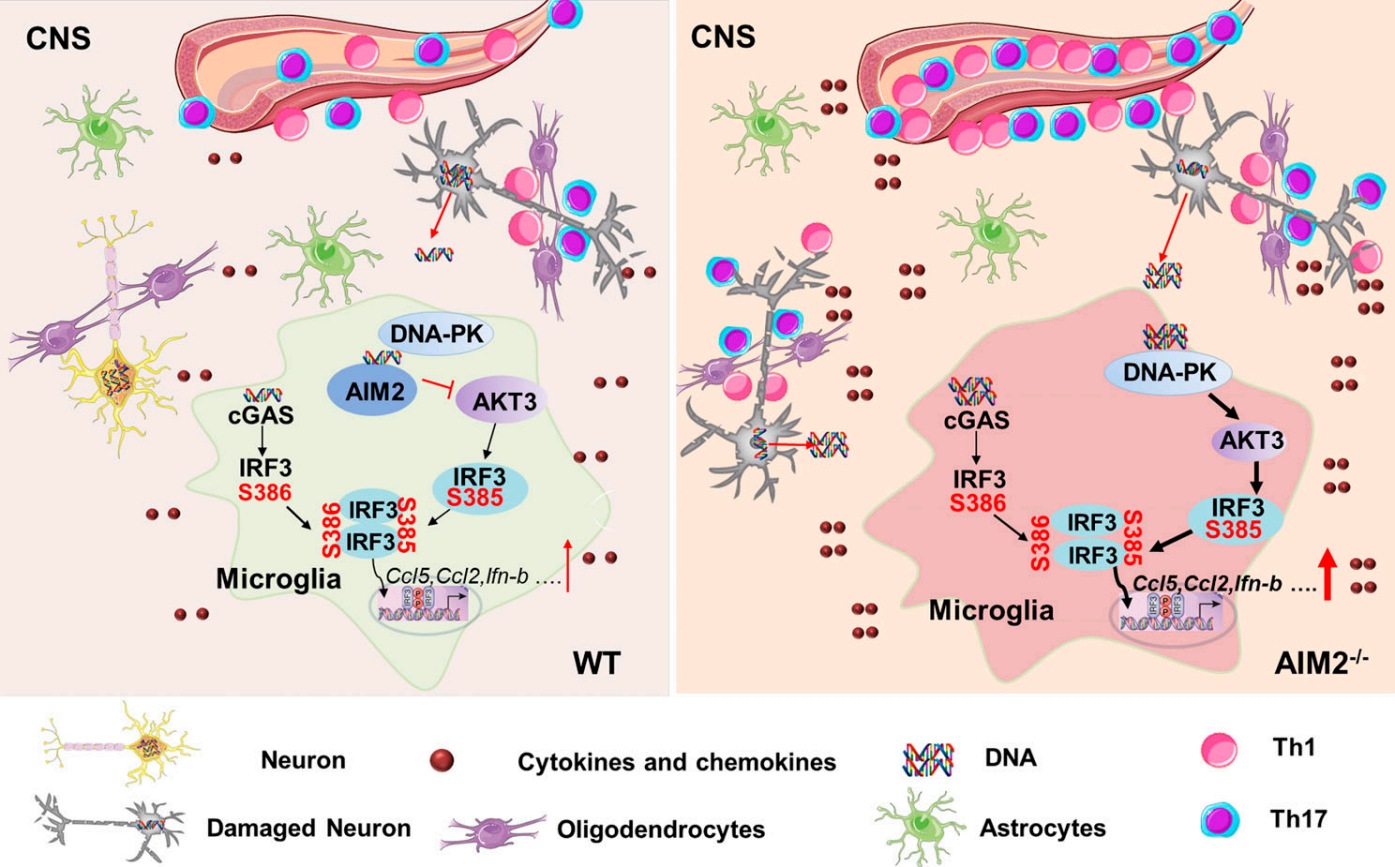
**Model for AIM2 function in microglia as a negative regulator of EAE.** The damaged double-stranded DNA released by dying nerve cells activates both cGAS and DNA-PK pathway in microglia. On one hand, cGAS senses double-stranded DNA to activate its downstream signaling effector STING via the production of cyclic guanosine monophosphate–adenosine monophosphate. Upon activation, STING recruits TBK1, which phosphorylates IRF3 at Ser386. At the same time, dsDNA activates DNA-PK to phosphorylate AKT3, which enhances IRF3 Ser385 phosphorylation. The phosphorylation of IRF3 at Ser385 facilitates STING-TBK1 to induce IRF3 Ser386 phosphorylation. The phosphorylation of Ser386 and Ser385 is required for IRF3 dimerization, which promotes IRF3 Ser396 phosphorylation and translocates to the nucleus to activate transcription of genes encoding antiviral inflammatory cytokines. In this process, AIM2 can interact with DNA-PK and blocks the binding of DNA-PK with AKT3, which suppresses AKT3 activation and downstream IRF3 Ser385 phosphorylation. Such effect inhibits cGAS and DNA-PK comediated-IRF3 activation and transcription of inflammatory cytokines. Thus, AIM2 in microglial cells negatively regulates the DNA-PK–AKT3 axis to inhibit neuroinflammation synergistically induced by cGAS and DNA-PK, thereby protecting against the onset of EAE.

In summary, our study describes an unexpected regulatory role of AIM2 in the development of EAE. We propose a model in which AIM2 can interact with DNA-PK to block the binding of DNA-PK with AKT3, which suppresses AKT3 activation and downstream IRF3 Ser385 phosphorylation. This interaction limits cGAS and DNA-PK IRF3 activation and transcription of inflammatory cytokines. Thus, AIM2 in microglial cells negatively regulates the DNA-PK–AKT3 axis to inhibit neuroinflammation and protect against the onset of EAE (Fig. 8). Our study uncovers an entirely new regulatory mechanism by which AIM2 modulates neuroinflammation and neurodegenerative diseases.

## Materials and methods

### Mice

Female mice with the C57BL/6 background were used in this study. The *AIM2*^−/−^ and *ASC*^−/−^ mice were a gift from Dr. V. Dixit (Genentech, South San Francisco, CA). The *cGAS*^−/−^ mice were from The Jackson Laboratory. The *AIM2*^fl/fl^ mice were generated using conditional gene targeting methods by Biocytogen. Aim2 conditional KO mice were generated by the CRISPR/Cas9-based approach. Briefly, two single guide RNAs (sgRNAs) were designed by a CRISPR design tool from the Feng Zhang lab (http://crispr.mit.edu; Platt et al., 2014) to target either a region upstream or downstream of exon 5, and then were screened for on-target activity using the Universal CRISPR Activity Assay (Biocytogen). To minimize random integrations, we employ a circular donor vector. The gene-targeting vector containing 5′ homologous arm, target fragment (exon 5), 3′ homologous arm was used as a template to repair the double-strand breaks generated by Cas9/sgRNA. The two LoxP sites were precisely inserted in both sides of the target fragment of the Aim2 gene. T7 promoter sequence was added to the Cas9 or sgRNA template by PCR amplification in vitro. Cas9 mRNA, targeting vector, and sgRNAs were coinjected into the cytoplasm of one-cell-stage fertilized C57BL/6N eggs. The injected zygotes were transferred into oviducts of Kunming pseudopregnant females to generate F0 (numerous founder 0) mice. F0 mice with the expected genotype confirmed by tail genomic DNA PCR and sequencing were mated with C57BL/6N mice to establish germline-transmitted F1 heterozygous mice. F1 heterozygous mice were genotyped by tail genomic PCR, Southern blot, and DNA sequencing. AIM2 floxed mice were crossed with *Cx3cr1*-Cre (The Jackson Laboratory) to generate mononuclear phagocyte–conditional AIM2 KO mice (*AIM2*^fl/fl^ *Cx3cr1*-Cre) or with *GFAP*-Cre (The Jackson Laboratory) to generate astrocyte-conditional AIM2 KO mice (*AIM2*^fl/fl^ *GFAP*-Cre). To generate the microglia-conditional AIM2 KO mice, AIM2 floxed mice were crossed with *Cx3cr1*-CreERT2-EYFP mice (The Jackson Laboratory), and then the mice were i.p injected with 3 mg tamoxifen (T5648; Sigma-Aldrich) dissolved in 200 µl corn oil (C8267; Sigma-Aldrich) for five consecutive days to induce the expression of Cre recombinase. After 6 wk, the tamoxifen-induced mice were microglia-conditional AIM2 KO mice, and these mice were used for the EAE study. All mice were kept in a barrier facility, and all animal experiments were conducted in accordance with the procedure approved by the Ethical Review Committee for Laboratory Animal Welfare of Nanjing Medical University.

### Antibodies and reagents

Antibodies to AIM2 (13095s), p-IRF3-S396 (4947s), IRF3 (4302s), p-TBK1 (5483s), TBK1 (3013s), p-AKT1 (9018s), AKT1 (2938s), p-AKT2 (8599s), AKT2 (3063s), p-AKT (4060P), and AKT3 (14982s) were purchased from Cell Signaling Technology. Antibodies to p-IRF3-S385 (D151514) were purchased from Sangon Biotech. Anti–ionized calcium binding adapter molecule 1 (Iba1; 019–19741) was from Wako. Anti-Apoe (ab1906) and anti-γH2AX (ab11174) were from Abcam. Anti-CD11b (101204) was from Biolegend. Anti-AKT (21054) was from SAB. Antibodies to actin (A1978) and DNA-PK (SAB4502385) were purchased from Sigma-Aldrich. Anti–CD45-FITC (30-F11,11-0451-82), anti–CD45-Alexa Fluor 700 (30-F11,56–0451-82), anti–CD8a-PE (53–6.7,12-0081-83), anti–CD11b-APC (M1/70,17-0112-82), anti–IL17-PE (eBio18B7,12-7177-81), anti–IFNγ-PerCP-Cyanine5.5 (XMG1.2,85-45-7311-82), anti–IFNγ-APC (XMG1.2,17-7311-82), and Fixable Viability Dye (FVD) eFluor 506 were from eBioscience. Anti–CD4-APC-Cy7 (GK1.5,100414) was from Biolegend. Pertussis toxin (#180) was from List Biological Laboratories. *Mycobacterium tuberculosis* H37Ra (231141) was from BD. Incomplete Freund’s adjuvant (F5506) was from Sigma-Aldrich. MOG35-55 peptide (residues 35–55, Met-Glu-Val-Gly-Trp-Tyr-Arg-Ser-Pro-Phe-Ser-Arg-Val-Val-His-Leu-Tyr-Arg-Asn-Gly-Lys) was synthesized by Sangon Biotech (Shanghai).

### Induction and assessment of EAE

To induce type A EAE, MOG_35-55_ peptide (300 µg per mouse) was emulsified with complete Freund’s adjuvant (200 µg *M. tuberculosis* H37Ra [Mtb] per mouse), and then subcutaneously injected in the flanks and neck of mice on day 0 for one immunization. Pertussis toxin (250 ng per mouse) was applied intravenously on days 0 and 2 after immunization. The type B EAE was induced as described above except that *M. tuberculosis* was administrated at 400 µg per mice. Mice were assessed daily for clinical signs of EAE in a blinded fashion. EAE score was evaluated as follows: 0.5, partial tail paralysis; 1, tail paralysis; 1.5, reversible corrective reflex impairment; 2, corrective reflex impairment; 2.5, one hindlimb paralysis; 3, both hindlimbs paralysis; 3.5, both hindlimbs paralysis and one forelimb paralysis; 4, hindlimb and forelimb paralysis; and 5, death.

### Bone marrow chimeras

The recipient mice were subjected to lethal-dose irradiation (10 Gy), and 1 d later, bone marrow cells (10 × 10^6^) derived from the tibiae and femurs of donor mice were i.v. injected into lethally irradiated mice. Under these conditions, the radio-resistant CNS-resident cells would be retained, whereas bone marrow and peripheral immune cells would be eliminated and replaced by bone marrow cells from donor mice. After 8 wk, the chimeric mice were then subjected to EAE induction.

### Histological analysis

All spinal cord tissue sections used here were 5 µm thick. For paraffin-embedded tissue, spinal cords collected from PBS-perfused mice were fixed in 4% paraformaldehyde overnight. Sections were stained with H&E for evaluation of leukocyte infiltration or with LFB for assessing demyelination. Histology was scored in a double-blinded fashion as followed: 0, no inflammatory cell infiltration and no demyelination; 1, slight inflammatory cell infiltration or demyelination observed; 2, moderate inflammatory cell infiltration or demyelination in several spots; and 3, substantial inflammatory cell infiltration and large area of demyelination.

### RNA in situ hybridization

Spinal cord tissue samples were fixed in formalin for 48 h, embedded in paraffin, and cut into 6-µm sections. In situ hybridization was performed according to the manufacturer’s instructions (G3017; Servicebio). Probes recognizing *Aim2* RNA (NM_001013779) were multiplexed with probes recognizing *DNAPK* RNA (NM_011159).

### Immunofluorescence staining

Tissue sections were incubated at 4°C overnight with primary antibody to IBA1, APOE, and CD11b. Slides were then incubated with indicated secondary antibodies. The nuclei were counterstained with DAPI (Sigma-Aldrich). Slides were dried and mounted using ProLong Antifade mounting medium (Beyotime Biotechnology). Slides were visualized by a Nikon 50i fluorescent microscope.

### Isolation of CNS immune cells and FACS analysis and sorting

For preparation of immune cells, brains and spinal cords from MOG35-55–immunized mice were excised and digested at 37°C with DNase I (10 U/ml; Roche) and collagenase type IV (0.5 mg/ml; Sigma-Aldrich) in RPMI 1640 under agitation (200 rpm) conditions for 60 min. Single-cell suspensions were obtained by grinding through a 70-µm cell strainer. Subsequently, homogeneous cell suspensions were centrifuged over the Percoll density gradient (GE Healthcare) and separated by collecting the interface fractions between 37 and 70% Percoll. Mononuclear cells were isolated from the interface. After intensive washing, single-cell suspensions were stained with FVD eFluor 506, anti-CD45, anti-CD4, anti-CD8, and anti-CD11b for FACS analysis. For intracellular cytokine staining, cells were stimulated with phorbol 12-myristate 13-acetate (Multi Sciences), ionomycin (Multi Sciences), and brefeldin A (Invitrogen) for 4 h of culture. Cells were fixed and permeabilized with the Intracellular Fixation & Permeabilization Buffer Set (eBioscience) and then subjected to cytokine staining flow cytometry analyses. All flow cytometry was performed on an Attune NxT flow cytometer (Thermo Fisher Scientific), and data were analyzed by FlowJo 10.0.7 software. For FACS sorting, single cell suspensions were stained with FVD eFluor 506, anti-CD45, and anti-CD11b and sorted on a BD FACS Aria.

### Primary microglia culture

Cerebral cortices from neonatal mice age 1–3 d were collected and carefully stripped of their meninges and the blood vessels. Following dissection, the tissues were digested with 0.25% trypsin–EDTA and washed in HBSS containing FBS. Then the single-cell suspensions were obtained by passing through a cell strainer (70 µm). The cell suspensions were seeded into poly-D-lysine–precoated flasks and cultured in DMEM/F12 supplemented with 10% FBS at 37°C and 5% CO_2_. Medium was replaced every 4–5 d. After 10–14 d, microglia were separated from the underlying astrocytic layer by gentle shaking of the flask, plated overnight in poly-D-lysine–precoated plates, and transfected with Lipofectamine 2000 (2 µl/ml)–complexed poly(dA:dT) (1 µg/10^6^ cells). For the DNA-PK inhibition experiment, the microglia were primed with 1 nM NU7441 (DNA-PK inhibitor) for 1h before poly(dA:dT) transfection. The conditioned media were collected and measured for cytokine production by ELISA, and the cells were collected for cytokine gene expression by RT quantitative PCR (RT-qPCR) or protein activation by Western blotting.

### siRNA-mediated gene silences in microglia

Primary microglia were plated in poly-D-lysine–precoated plates and were transfected with siRNA using Lipofectamine RNAiMAX (Invitrogen) according to the manufacturer’s guidelines. The siRNA sequences were: siAKT1-1 (sense: 5′-CCA​UGA​ACG​AGU​UUG​AGA-3′; antisense: 5′-GGU​ACU​UGC​UCA​AAC​UCA​U-3′), siAKT1-2 (sense: 5′-CUU​CCU​CCU​CAA​GAA​CGA​U-3′; antisense: 5′-GAA​GGA​GGA​GUU​CUU​GCU​A-3′), siAKT2-1 (sense: 5′-GGA​GGU​AGC​UGU​CAA​CAA​G-3′; antisense: 5′-CUU​GUU​GAC​AGC​UAC​CUC​C-3′), siAKT2-2 (sense: 5′-GCA​AAG​AGG​GCA​UCA​GUG​A-3′; antisense: 5′-UCA​CUG​AUG​CCC​UCU​UUG​C-3′); and siAKT3 (sense: 5′-GCU​CAU​UCA​UAG​GCU​AUA​A-3′; antisense: 5′-UUA​UAG​CCU​AUG​AAU​GAG​C-3′). The siRNA and negative control siRNA were from GenePharma.

### RT-qPCR

Total RNA was extracted by using TRIzol reagent (Life Technologies) and subjected to cDNA synthesis. RT-qPCR was performed using SYBR Green Supermix (Vazyme). The expression of a single gene was calculated by a standard curve method and standardized to the expression of *Hprt*. The primers used are listed in Table 1.

**Table 1.** Primers used for RT-qPCR

	S	AS
*Siglec1*	5′-CAG​GGC​ATC​CTC​GAC​TGT​C-3′	5′-GGA​GCA​TCG​TGA​AGT​TGG​TTG-3′
*Axl*	5′-ATG​GCC​GAC​ATT​GCC​AGT​G-3′	5′-CGG​TAG​TAA​TCC​CCG​TTG​TAG​A-3′
*Cybb*	5′-TGT​GGT​TGG​GGC​TGA​ATG​TC-3′	5′-CTG​AGA​AAG​GAG​AGC​AGA​TTT​CG-3′
*Clec7a*	5′-GAC​TTC​AGC​ACT​CAA​GAC​ATC​C-3′	5′-TTG​TGT​CGC​CAA​AAT​GCT​AGG-3′
*Gpr34*	5′-CTC​AGG​AGT​GCC​AAA​TGT​CAC-3′	5′-GCC​CAG​AAA​TAC​ATA​GAG​GGC​AA-3′
*Tgfbr1*	5′-TCT​GCA​TTG​CAC​TTA​TGC​TGA-3′	5′-AAA​GGG​CGA​TCT​AGT​GAT​GGA-3′
*Cxcl10*	5′-AAG​TGC​TGC​CGT​CAT​TTT​CTG-3′	5′-TTC​CCT​ATG​GCC​CTC​ATT​CTC-3′
*Il-6*	5′-CTT​GGG​ACT​GAT​GCT​GGT​GAC-3′	5′-GCC​ATT​GCA​CAA​CTC​TTT​TCT​C-3′
*Tnf*	5′-TAC​TGA​ACT​TCG​GGG​TGA​TCG-3′	5′-TCC​TCC​ACT​TGG​TGG​TTT​GC-3′
*Ccl5*	5′-GAC​ACC​ACT​CCC​TGC​TGC​TT-3′	5′-ACA​CTT​GGC​GGT​TCC​TTC​G-3′
*Irf7*	5′-CAG​CAC​AGG​GCG​TTT​TAT​CTT-3′	5′-TCT​TCC​CTA​TTT​TCC​GTG​GC-3′
*Fcrsl*	5′-CTT​CTG​GTC​TTC​GCT​CCT​GTC-3′	5′-ATG​GTG​TAG​CTT​GAA​GCA​CTG-3′
*Ddx58*	5′-CAC​ATT​TGC​GGA​AAT​ACA​ACG-3′	5′-TGC​TGC​TTC​TCG​GAC​ATC​G-3′
*Ifit1*	5′-TGC​TTT​GCG​AAG​GCT​CTG​A-3′	5′-AAT​CTT​GGC​GAT​AGG​CTA​CGA​C-3′
*Oasl2*	5′-TTG​TGC​GGA​GGA​TCA​GGT​ACT-3′	5′-TGA​TGG​TGT​CGC​AGT​CTT​TGA-3′
*Stat1*	5′-GTC​ACA​GTG​GTT​CGA​GCT​TCA​G-3′	5′-CGC​AAA​CGA​GAC​ATC​ATA​GGC-3′
*Hprt*	5′-GTC​CCA​GCG​TCG​TGA​TTA​GC-3′	5′-TGG​CCT​CCC​ATC​TCC​TTC​A-3′
*Apoe*	5′-CTG​ACA​GGA​TGC​CTA​GCC​G-3′	5′-CGC​AGG​TAA​TCC​CAG​AAG​C-3′

AS, antisense; S, sense.

### Immunoprecipitation and immunoblot analysis

Primary microglia were transfected with Lipofectamine 2000 (2 µl/ml)–complexed poly(dA:dT) (1 µg/10^6^ cells). Cells were collected in NP-40 lysis buffer (20 mM Tris-HCl, pH 7.4, containing 150 mM NaCl, 0.5% [vol/vol] IGEPAL, 10% [wt/vol] glycerol, 50 mM NaF, 1 mM Na_3_VO_4_, 1 mM dithiothreitol, 1 mM phenylmethylsulphonyl fluoride, and complete protease inhibitor cocktail [Sigma-Aldrich]), followed by incubation for 40 min at 4°C. The lysates were centrifuged for 15 min at 14,000 rpm for removal of cell debris and nuclei. Supernatants were incubated with the indicated antibody overnight at 4°C. Then an aliquot (40 µl) of protein A/G-agarose was added to each sample, followed by incubation for 4 h at 4°C. The beads were washed three times with 500 ml lysis buffer. An aliquot (50 µl) of SDS-PAGE sample buffer was added to the beads to separate protein. Samples were resolved by SDS-PAGE, transferred to nitrocellulose membranes, and analyzed by immunoblot with the appropriate antibodies.

### ELISA

Primary mouse microglia were stimulated as indicated. Conditioned media were collected and measured for levels of TNF-α (DY410), IL-6 (DY406), CCL5 (DY478), CXCL10 (DY466), and CCL2 (DY479) according to the manufacturer’s instructions (R&D Systems). IFN-β ELISA capture antibody (sc-57201) was from Santa Cruz; IFN-detection antibody (32400–1) and standard protein (12400–1) were from R&D Systems. The level of IFN-β was assayed by an in-house sandwich ELISA system as previously described (Li et al., 2019a).

### Microarray analysis

For microarray analysis, CD45^low^CD11b^+^ microglial cells were isolated from CNS of WT and *AIM2*^−/−^ mice on day 18 after immunization by sorting on a BD FACS Aria. Total RNA was extracted with Trizol reagent. Samples were analyzed by Beijing Cnkingbio Biotechnology with Affymetrix Mouse Clariom S Array, and all arrays were scanned by using Affymetrix GeneChip Command Console, which was installed in a GeneChip Scanner 3000 7G. The data were analyzed with Robust Multichip Analysis algorithm using Affymetrix default analysis settings and global scaling as a normalization method. Values presented are log2 Robust Multichip Analysis signal intensity. Differentially expressed genes were identified based on the Student’s *t* test for comparison of the two groups. The threshold set for up- and down-regulated genes was a fold change >1.2 and a P value <0.05. KEGG pathway analysis and GO analysis were performed using the R package, using significantly differentially expressed genes (P < 0.05) as target genes. Gene network analyses were done by the Beijing Genomics Institute in-house customized data mining system called Dr. Tom. GSEA was performed as previously described (Subramanian et al., 2005) using the Gene Ontology Biological Process Database. Raw data files and processed files have been deposited in GEO under accession no. GSE151636.

### Statistical analyses

The data were analyzed by GraphPad Prism 7.0 software and are presented as mean ± SEM. The statistics were analyzed by using a two-tailed unpaired *t* test for two groups and a multiple unpaired *t* test for multiple groups. P values are *, P < 0.05; **, P < 0.01; and ***, P < 0.001.

### Online supplemental material

Fig. S1 shows the AIM2 conditional KO mice strategy. Fig. S2 shows that AIM2 deficiency in microglia but not in astrocytes promotes the development of EAE. Fig. S3 shows that AIM2 deficiency in microglia promotes the neuroinflammation of EAE. Fig. S4 shows that AIM2 negatively regulates the DNA-PK–AKT3 in microglia to control inflammation. Fig. S5 shows that AIM2 protects against cGAS-mediated neuroinflammation and AIM2 expression is increased in the brain tissues of MS patients.
